# Harmonization of the fastest and densest responses reflects humanlike reaction time in mice

**DOI:** 10.3389/fnins.2025.1501374

**Published:** 2025-01-29

**Authors:** Chan Hee Kim

**Affiliations:** Department of Physiology and Neuroscience, Dental Research Institute, Seoul National University School of Dentistry, Seoul, Republic of Korea

**Keywords:** reaction time, stimulus duration, head entry, mice, behavior

## Abstract

**Introduction:**

Reaction time (RT) is important for evaluating delayed latency in behavior. Unlike humans, whose RT usually reflects a one-to-one stimulus–response relationship, the RT of animals can show two peaks representing the fastest and densest responses in the response distribution due to multiple responses per trial and can be further delayed depending on stimulus duration.

**Methods:**

Stimulus duration was controlled to investigate whether these two peak latencies align to form a single RT. Sound cues lasting 10, 5, and 2 s, each associated with a food reward of condensed milk, were tested in three groups of 24 mice using delay conditioning paradigm. The frequency and latency of responses, along with basic indices such as accuracy, were analyzed.

**Results:**

In delay conditioning experiments using sound cues of 10, 5, and 2 s, the 2 s group exhibited the strongest positive correlations between the two peaks, as well as between the number of responses and accuracy rate, suggesting a coupling of the fastest and densest responses and a one-to-one relationship between stimulus and response.

**Discussion:**

Based on these findings, I propose harmonizing the two peaks, elicited by stimuli that induce prompt and minimal responses, as a criterion for designing animal experiments to better mimic humanlike RT.

## Introduction

Reaction time (RT), also known as response time (Schouten and Bekker, 1967; Luce, 1986; Baayen and Milin, 2010; Welford et al., 1980), is essential in behavior analysis. In human and animal studies, terms such as reaction speed (Stefanics et al., 2010; Khamechian et al., 2019) and response latency (Etzel and Wright, 1964; Mehraei et al., 2016) are utilized interchangeably with RT. RT is typically evaluated within a specific time frame for each trial (Koelsch et al., 2002), along with an estimate of its time point (Poulton, 1950; Butz and Hoffmann, 2002). In human studies, researchers provide clear instructions to human subjects before experiments to ensure RT sensitivity. Human subjects are occasionally asked to respond quickly (Altieri and Townsend, 2011; Eshel and Roiser, 2010). However, due to communication limitations, instructing animals to respond quickly may be impractical. Even with clear instructions and extended training in an experimental paradigm, animals may deliberately delay their responses if the interval between stimulus onset and the reward is sufficiently long. Furthermore, their behavior during a trial, which includes repeated actions such as licking, nose poking, and lever pressing, complicates the interpretation of RT-like measures. Animal behavioral responses have focused on whether animals sufficiently and successfully respond in experimental paradigms, and then the repeated responses have been considered part of animals’ nature. However, these repeated responses are all RTs in the context of animal experiments, but it is impossible to discern which of the repeated responses are comparable to human data. The current study proposes theoretical criteria for determining humanlike RT from animal data.

**Figure 1:**
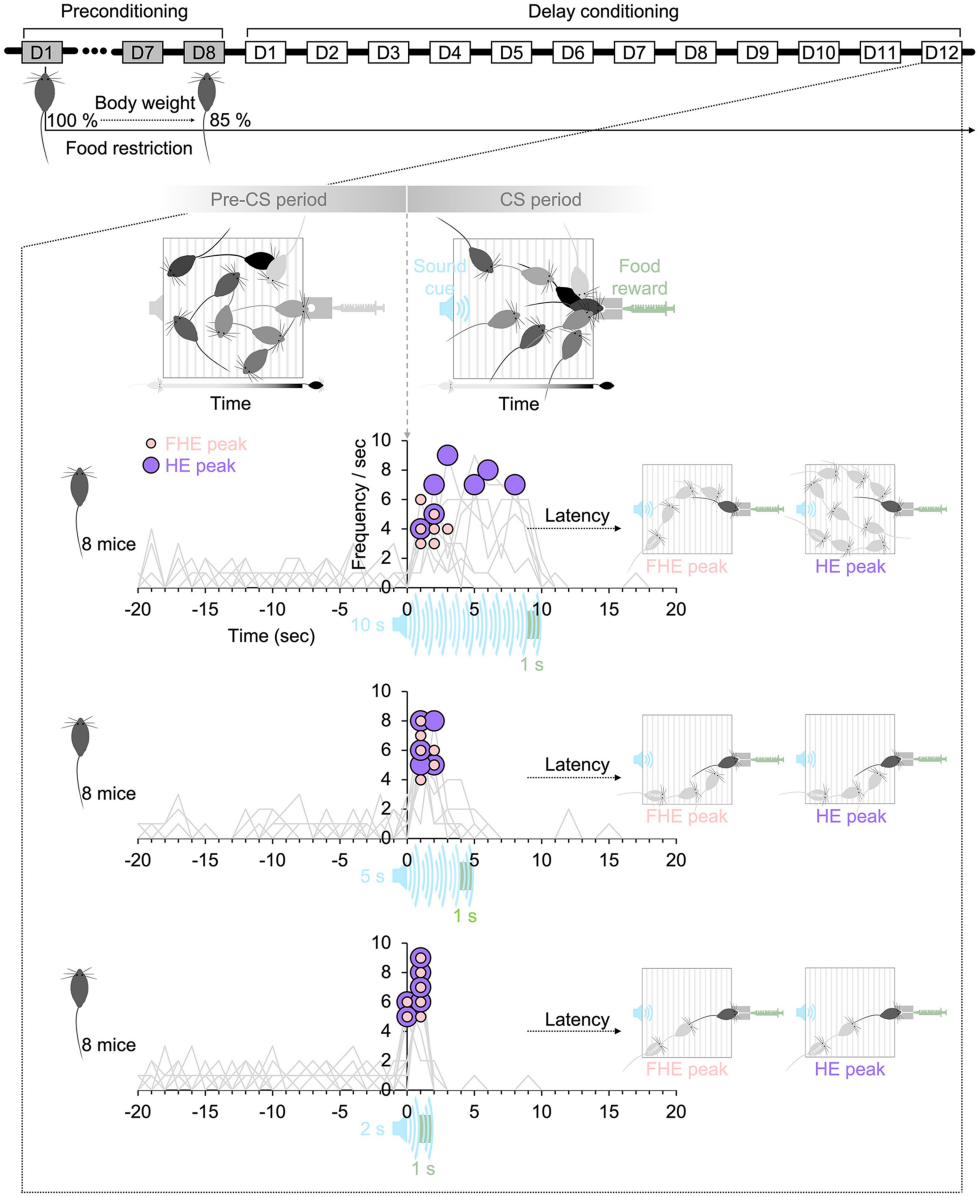
Experimental paradigm. The mice in all groups lost up to 85% of their body weight by restricting their food intake before delay conditioning. During the CS period, the duration of the sound cue followed by food reward varied among the 10-, 5-, and 2 s groups. On the final training day (D12), the HE peak latency in the CS period after the onset of the sound cue was higher than that in the pre-CS period before the onset of the sound cue. The FHE peaks occurred before the HE peaks in the 10 s group, whereas in the 5 and 2 s groups, the two peaks were nearly synchronized. The latencies of the two peaks show how mice in each group respond differently to sound cues with varying durations. HE, head entry; FHE, fastest HE; D, training day; CS, conditioned stimulus.

The stimulus–response relationship per trial may not be one-to-one in animal studies as in human studies (Brewer, 2011; Grice et al., 1982). Animals respond even in a moment without a sound cue or reward. In the experimental paradigm of classical conditioning (Figure 1), the animals’ responses are not zero in the time window from −20 s to 0 s; instead, they increase dramatically during the period with a sound cue and reward after 0 s. Thus, a single timepoint of RT for each trial, commonly observed in human studies (Kim et al., 2021; Kim et al., 2014), may be comparable to the fastest response in repeated animal responses (Supplementary Figure 1A). However, the frequency of repeated responses, such as head entry (HE) into the food cup, is critical for assessing the animals’ behavioral performance (Harris and Carpenter, 2011; Austen and Sanderson, 2020; Jennings and Kirkpatrick, 2006). During the training period, repeated HEs for repeated stimulations gradually become the densest point and form a peak in the HE distribution, which reflects the behavioral habituation and sensitization of subjects (Groves and Thompson, 1970; de Boer et al., 1976; Thompson, 2009; Cevik, 2014; McSweeney et al., 1996; McSweeney and Murphy, 2009). Therefore, in addition to the HE peak latency, the fastest HE (FHE) and its peak may be present within the HE distribution (Figure 1 and Supplementary Figures 1, 2). Fundamentally, the time points for RTs in animal data would not be a single latency but rather a subset of both components of FHE and HE peak latencies. The coexistence of two distinct components may raise an issue that requires the identification of a true RT in the data. Considering a one-to-one relationship like that in humans, the FHE peak after stimulus onset would be the true RT. In contrast, the HE peak would have reliability as the true RT owing to its higher frequency. Nevertheless, if the FHE and HE peaks coincide, it indicates a coupling of the fastest and densest responses. This is an ideal option that is closer to humans’ RT.

Subtle methodological differences exist between animals and humans owing to the distinct behavioral characteristics and experimental procedures, even when directly comparing their RTs (Bale et al., 2017; Esteves et al., 2021; Johnson et al., 2017). For instance, in a task to detect a sound cue, human subjects sit down at a desk and press a mouse whenever they detect the cue. Food restriction before the experiment and reward per trial are not essential for human subjects’ click behavior. They do not click to receive the reward, except in a specific paradigm (Balodis et al., 2012; Marco-Pallares et al., 2008). The current experimental paradigm to test the humanlike RT of animals was selected on this basis. The purpose of this study is not to propose new equipment or experimental paradigms to correct and reinforce animal behavior but rather to propose quantitative indicators that can serve as a foundation for designing experimental paradigms to compare human and animal behavior. To eliminate additional factors beyond the basic concept of learning food–reward associations and to observe the mice’s natural behaviors and the underlying nature of their responses to a sound cue, the experimental paradigm was based on classical conditioning, which also influences operant conditioning. The experimental protocol did not include rewards or punishment for missed responses. Since both the frequency and latency of behavioral responses are critical factors explored in relation to animal behavior in numerous studies, experimental paradigms, such as the use of retractable levers to prevent repetitive behavior, were not considered. Also, the animals’ heads or bodies were not restrained during the experiment. During 12 days of delay conditioning (Clark and Squire, 1998), the freely moving mice learned the food–reward association and were trained to respond when they detected a sound cue (Figure 1), similar to human subjects.

RT can be influenced by various experimental conditions. This study aimed to define the effects of a temporal factor in experimental design that could combine the two different latencies of FHE, and HE peaks into a single cohesive unit of RT rather than treating them as separate entities. I thought that the key factor was the duration of a conditioned stimulus (CS) associated with an unconditioned stimulus (US). The shorter CS may reduce unnecessary repeated responses and encourage prompt reactions, improving the frequency and latency of responses. Indeed, many studies have explored the effect and significance of the CS–US interval (or CS duration) based on tests with various CS durations (Harris and Carpenter, 2011; Austen and Sanderson, 2020; Jennings and Kirkpatrick, 2006; Johnson, 2018; Rosas and Alonso, 1996; Schwarz-Stevens and Cunningham, 1993; Meltzer, 1988; Meltzer and Brahlek, 1970; Delamater and Holland, 2008; Holland, 1980; Thrailkill et al., 2020). However, the specific durations of the stimulus vary depending on the apparatus and study paradigm. Thus, an absolute value cannot be used to determine the appropriate duration for designing an experiment. To investigate this, I tested separate groups of C57BL/6NCrljOri mice (eight male mice for each group) with sound cues of different CS durations (white noise of 10, 5, and 2 s) (Figure 1). I hypothesized that the latency and frequency of the FHE and HE peaks would vary depending on the CS durations. If the FHE and HE peak latencies are synchronized, their frequencies would also align into a single peak because the relationship between latency and frequency can be interdependent. Moreover, I hypothesized that the optimal CS duration could be predicted based on the relationship between the two peaks. Thus, using white noise and condensed milk, I recorded the HEs of freely moving mice in an operant chamber based on delay conditioning.

## Methods

### Subjects

A total of 24 naïve male C57BL/6NCrljOri mice, aged 7 weeks old, were provided by the Experimental Animal Center at Seoul National Dental University. Eight mice were assigned to each group. Before the delay conditioning, each mouse was housed in its own cage and subjected to a food restriction schedule to maintain their weight at 85% of their free-feeding weight throughout the study. They had unlimited access to water. The mice were kept in cages in a room with a light–dark cycle of 12 h each (8 am–8 pm). Training began when the mice reached 8 weeks old and ended when they were 10 weeks old. The care and use of every animal were approved by the Seoul National University Institutional Animal Care and Use Committee (SNU IACUC).

### Apparatus

The experiments were conducted using operant chambers (interior dimensions: 21.6 × 17.8 × 12.7 cm; model EBV-307 W-CT, Med Associates), each equipped with a stainless steel grid floor consisting of 24 rods with a diameter of 0.32 cm (17.8 × 15.2 × 5.7 cm, model ENV-307 W-GF) and enclosed within MDF sound-attenuating cubicles (model ENV-022MD). A house light (28 V DC, 1000 mA, model ENV-315 W) remained on throughout each experiment. A sound cue of 75 dB (Tosun et al., 2016) was produced by a white noise amplifier (10–25,000 Hz, model ENV-325SW) and delivered through a cage speaker (model ENV-324 W). The noise produced by fans (model ENV-025F) in each cubicle was approximately 65 dB. Food reward, which consisted of condensed milk, was delivered into a single 0.5-cc stainless steel cup through a liquid pipe (model ENV-303LPHD) connected to a syringe pump (model PHM-100A-EURO). The HEs were counted whenever mice broke the detector beam (model ENV-303HDW) inside the liquid pipe. The experiments were conducted using the MED-PC V software (model SOF-735).

### Stimulus

The experiment included two stimuli: CS and US. The CS consisted of white noise cues with varying durations: 10, 5, and 2 s (Figure 1 and Supplementary Figure 3B). The selection of a CS duration of 10 s was influenced by literature (Austen and Sanderson, 2020; Thrailkill et al., 2020; Trott et al., 2022; Strickland et al., 2024) on CS duration. The 5 s and 2 s durations were determined using the HE and FHE peak latencies observed in the preliminary tests with a 10 s CS duration. The current data also observed the FHE and HE peak patterns (Supplementary Figure 3C). To ensure that the CS durations were within the ranges where mice could respond effectively, I examined the responses to the 10 s CS. I selected the 2 s duration based on the peak latency of FHE and the 5 s duration based on the peak latency of HE observed during the late training sessions. The US consisted of condensed milk. In accordance with the principles of delay conditioning, the offset timings of the US matched those of the CSs (Figure 1). Therefore, each US lasted 1 s and occurred between 9 and 10 s after the onset of the 10 s CS, 4–5 s after the onset of the 5 s CS, and 1–2 s after the onset of the 2 s CS.

### Procedure

The preconditioning phase lasted 8 days, during which the mice underwent food restriction to reduce their weight to 85% of baseline before the main experiment’s delay conditioning phase. They were also acclimated to handling the US. On the last day of the preconditioning phase, the mice had a session in the chamber without the CS but with the US. After preconditioning, the delay conditioning experiments were repeated daily for a total of 12 days (Figure 1). Each day, 10 trials were conducted during the experiment. The intertrial intervals (ITIs) were varied and were randomly set to 90, 180, or 270, based on a mean ITI of 240 and an ITI variance of 120 (Supplementary Figure 3A). The total experimental time per subject was about 1 h per day, including the preparation time. During the experiments, pumps were used to deliver condensed milk from syringes to food cups, with the pumps turned on during the US period. The US was delivered once over a 1-s interval at the end of the CS presentation, regardless of whether the mice responded. HE’s response was not required to trigger delivery.

Furthermore, the protocol did not include punishments for missed or premature responses, nor were rewards withheld in such cases. The mice were free to move around the chamber. In this study, the behavior directed toward the food cup to consume the reward food was referred to as “HE.” The timing of HEs to the food cup was recorded at a temporal resolution of 10 ms.

### Analysis

**Figure 2:**
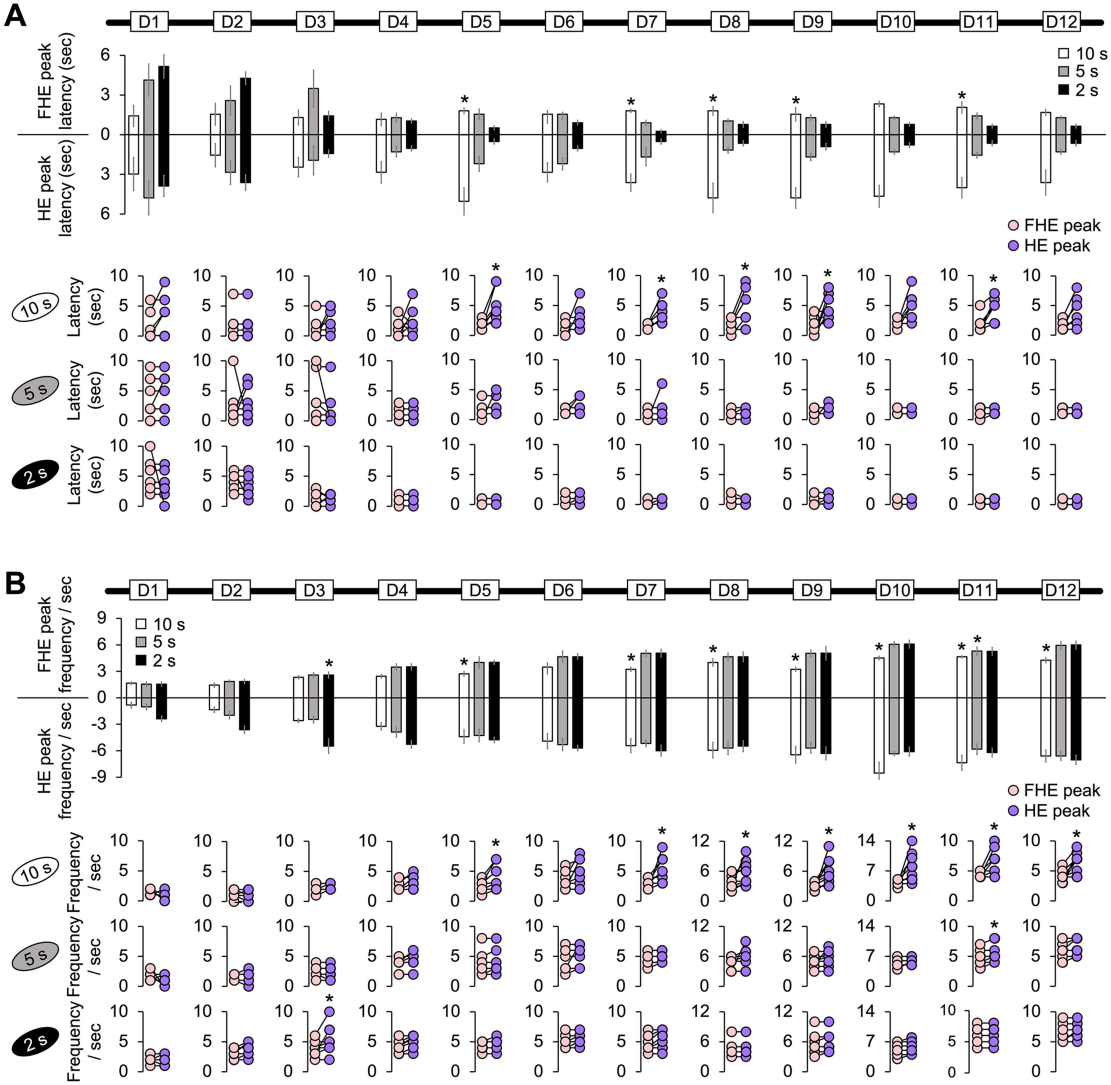
Difference between the FHE and HE peaks in latency and frequency. **(A)** Only the 10 s group exhibited significant differences in latencies between the FHE and HE peaks (Wilcoxon signed-ranks test, *p* < 0.05). The latency for the HE peak was greater than that for the FHE peak. However, no significance was observed in the 5 and 2 s groups (Wilcoxon signed-ranks test, *p* > 0.05). **(B)** The 10 s group showed significant differences in the FHE and HE frequencies in D5 and D7–D12 (Wilcoxon signed-ranks test, *p* < 0.05). The frequency of the HE peak was higher than that of the FHE peak. In the 2 s group, a significant difference was observed in D3. The 5 s group was only significant in D11. Supplementary Tables 1, 2 also provide details of the statistical analyses. *, *p* < 0.05. HE, head entry; FHE, fastest HE; D, training day.

**Figure 3:**
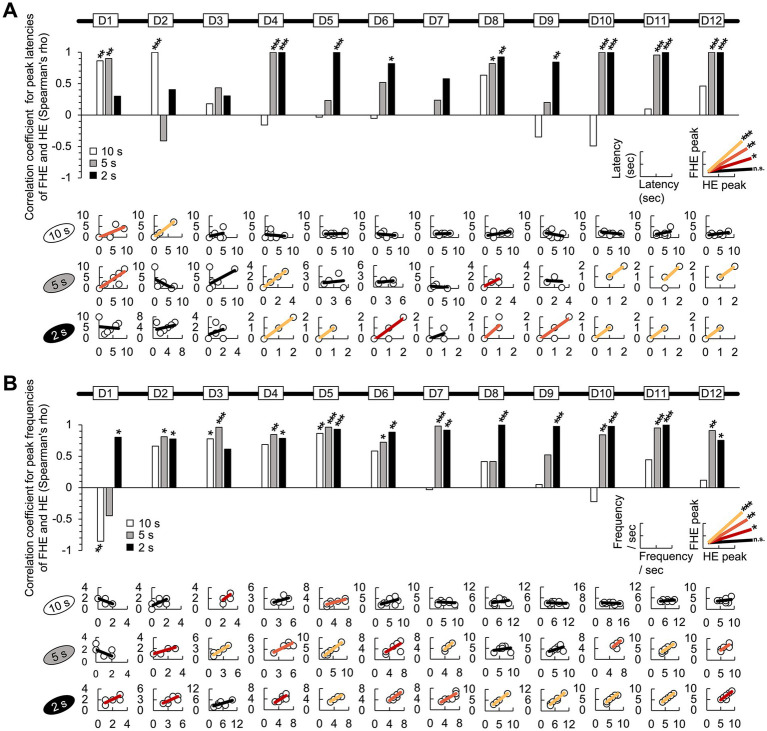
Correlation between the FHE and HE peaks in latency and frequency. **(A)** In the results of the Spearman test, the latencies of the FHE and HE peaks were strongly and positively correlated in later training days for the 2 s group (D4–D6 and D8–D12). Positive correlations were found in the later days of the 5 s group (D1, D4, D8, D10, D11, and D12) but only on D1 and D2 for the 10 s group. **(B)** Spearman test exhibited that the 2 s group exhibited strong positive correlations in the frequencies of the FHE and HE peaks across all 12 days, except for D3. Furthermore, significant positive correlations were observed in the 5 and 10 s groups. In the 5 s group, positive correlations were found on all days except for D1, D8, and D9. However, in the 10 s group, positive correlations were observed only during the early stages of training (D3 and D5). The nonparametric correlation was evaluated using the Spearman test. Supplementary Tables 3, 4 also provide details of the statistical analyses. *, *p* < 0.05; **, *p* < 0.01; and ***, *p* < 0.001. HE, head entry; FHE, fastest HE; D, training day.

**Figure 4:**
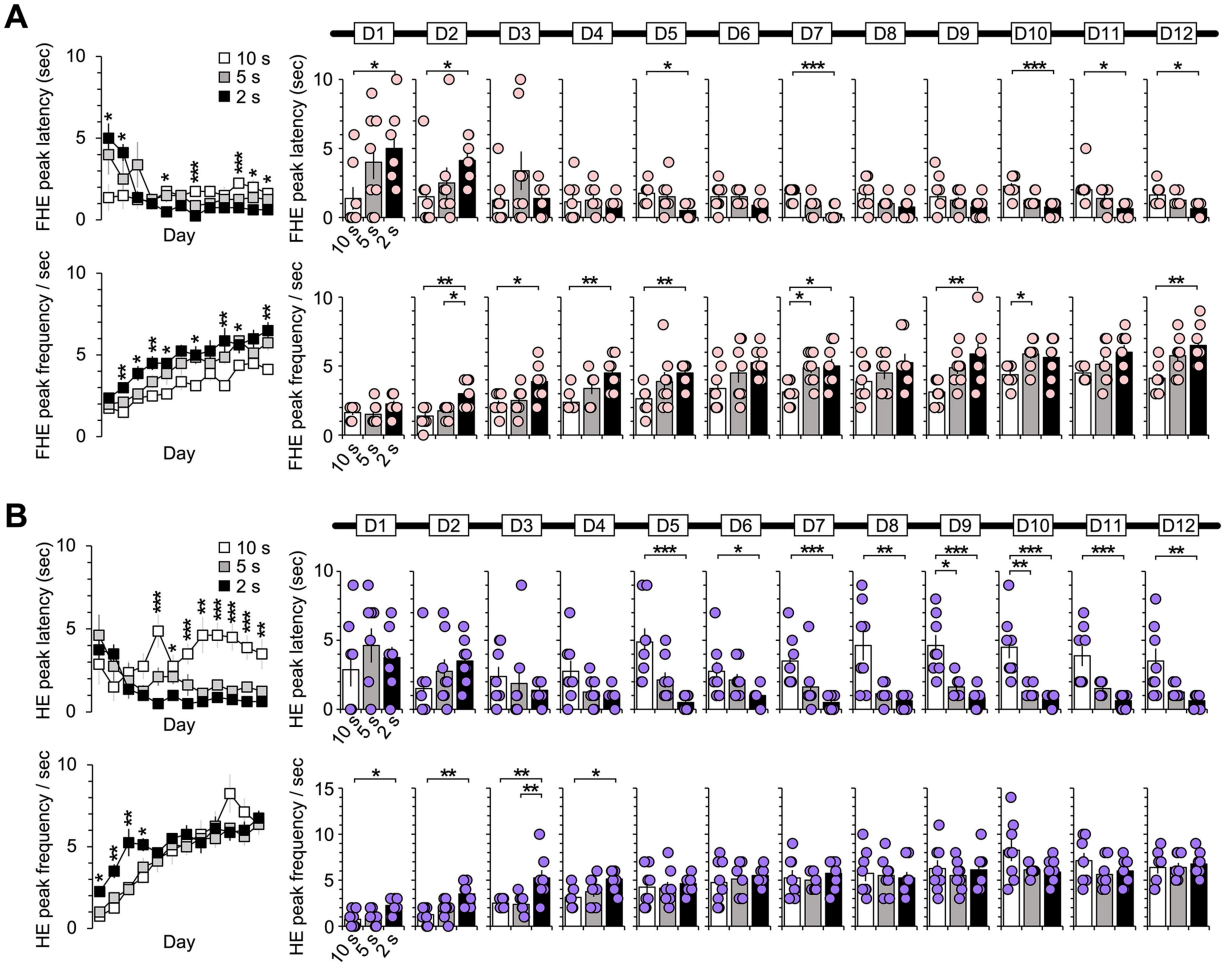
Difference between groups in the latency and frequency of the FHE and HE peaks. The one-way ANOVA results for the FHE and HE peak latency and frequency indicated a significant effect of group factors (10, 5, and 2 s). **(A)** The FHE peak exhibited a significant group effect across training days for both latency (D1, D2, D5, D7, D10, D11, and D12) and frequency (D2, D3, D4, D5, D7, D9, D10, and D12) (Kruskal–Wallis H test, *p* < 0.05). *Post hoc* analyses revealed significant differences between the 10 and 2 s groups or between the 10 and 5 s groups for both latency and frequency (Dunn’s test, *p* < 0.05, Bonferroni-corrected). **(B)** The one-way ANOVA results for the HE peak indicated a significant group effect for both latency (D5–D12) and frequency (D1–D4) (Kruskal–Wallis H test, *p* < 0.05). In the *post hoc* analysis, the latency of the 10 s group significantly differed from the other groups, starting with D5 (Dunn’s test, *p* < 0.05, Bonferroni-corrected). In the *post hoc* analysis for frequency, the 2 s group differed the most from the other groups from D1 to D4 (Dunn’s test, *p* < 0.05, Bonferroni-corrected). Supplementary Tables 5–8 also provide details of the statistical analyses. *, *p* < 0.05; **, *p* < 0.01; and ***, *p* < 0.001. Error bars denote 95% confidence intervals. HE, head entry; FHE, fastest HE; D, training day.

The data was analyzed in three groups of 24 mice, each with a duration of 10, 5, and 2 s. For the HE frequency analysis, the HEs were extracted from the CS and pre-CS time windows for 10 trials, and the number of HEs was normalized per s. I carefully selected the time window of the pre-CS period as an off-stimulus baseline to compare to the CS of the on-stimulus condition. Supplementary Figure 3D shows the details of pre-CS segmentation. In the analysis of HE accuracy, the presence or absence of HE during each CS duration per trial was calculated and converted to percentages (where one correct trial equates to 10%). The number of HE was calculated using the total number of HEs during each CS duration. The FHEs were extracted from the first HE in each trial, including the HEs (Supplementary Figure 1). To exclude the errors caused by too-prompt and too-delayed responses associated with mice’s heads put in the food cup before CS onset, I extracted a peak in all responses for both HE and FHE. The FHE and HE peak values between 0 s and 1 s after the CS onset were recorded as 1 in terms of latency (Supplementary Figures 1, 3). The maximum frequency value for the FHE peak per 1-s epoch was 10, the trial number per day, whereas the HE peak per 1-s epoch can be over 10 (Figures 2–4). The FHE peak represents a peak in the distribution of FHEs, while the HE peak corresponds to a peak in the distribution of HEs. Both peaks include frequency and latency values (Supplementary Figure 1). Accordingly, the latencies of the peaks did not include the incorrect HEs, as RT is typically based on correct answers (Kail, 1991; Novikov et al., 2017). The time windows for the estimation of the FHE and HE peaks were each set from 0 to 10 s after the onset of CS in all groups (Supplementary Figure 4). The baseline-corrected HE frequency and peak were calculated by subtracting the mean HE in the pre-CS period from the HE in the CS (Supplementary Figures 5, 6).

### Statistics

The statistical analysis showed that all data did not follow a normal distribution. Thus, nonparametric analyses were employed for all statistical procedures. Nonparametric Wilcoxon signed-rank tests between the FHE and HE peaks were conducted using the Wilcoxon signed-ranks test. The non-parametric correlations between each pair of the FHE and HE peaks and the number of HEs and accuracy were determined using the Spearman test. All three groups had a correlation coefficient value 1, suggesting that the FHE and HE peaks coincided (Supplementary Figure 3). Notably, converting a decimal to an integer during the extraction of the two peaks from HEs significantly affected the highest correlation coefficient values (Supplementary Figure 3). Nonparametric variances (ANOVAs) analysis was conducted using the Kruskal–Wallis H test. In addition, *post hoc* analyses for ANOVAs were conducted using Dunn’s test. The Bonferroni test adjusted the type I error caused by multiple comparisons between the three groups in Dunn’s test. MATLAB 9.12.0.2039608 (Math Works Inc., Natick, MA, USA) and SPSS 25.0 software (IBM, Armonk, NY, United States) were used to conduct all analyses.

## Results

In the data analysis, I conducted a detailed analysis of behavioral data at both the temporal and frequency levels to fully evaluate the impact of CS duration. The analysis encompassed several steps. First, I assessed the difference and correlation between the FHE and HE peaks to better understand their relationship. Second, I separately investigated the group differences in the FHE and HE peaks to better understand their characteristics and implications. Third, I examined classical components such as HE frequency during the CS and pre-CS periods and HE accuracy to confirm the effect of CS duration on subject performance.

Furthermore, I considered how shorter than longer CS durations affected the mice’s ability to reach the food cup and how CS duration affected the HE frequency. Based on these findings, I determined how the subjects’ performance was affected by the relationship between the FHE and HE peaks. Finally, I examined the correlation between the number of HEs for each trial and accuracy across each group to determine whether the relationship between HE responses and correctness reflected humanlike behavior. Based on these processes, I proposed a criterion for determining an appropriate CS duration to elicit a humanlike RT based on the alignment of the FHE and HE peaks.

### Difference between the FHE and HE peaks for each group

The results from the non-parametric Wilcoxon signed-rank test for all training days indicated that the 10 s group mostly exhibited differences in latency and frequency between the FHE and HE peaks. The significance of these findings varied by training days (D). Specifically, for latency, the HE peaks were longer than the FHE peaks on D5, D7, D8, D9, and D11 (Figure 2A and Supplementary Table 1; Wilcoxon signed-ranks test, *p* < 0.05 in all cases). Similarly, the frequencies of the HE peaks were higher than those of the FHE peaks on D5 and D7 through D12 (Figure 2B and Supplementary Table 2; Wilcoxon signed-ranks test, *p* < 0.05 in all cases). In the 2 s group, a significant difference was observed in D3. The 5 s group was only significant in D11. Except for the aforementioned results, no significant differences were observed in latency or frequency between the FHE and HE peaks (*p* > 0.05 in all cases).

### Correlation between FHE and HE peaks for each group

Based on the nonparametric Wilcoxon signed-rank test results, we employed the Spearman test to determine the correlation between the FHE and HE peaks. As expected, the FHE and HE peak latencies of the 2 s group were strongly and positively correlated during the later training days (D4–D6 and D8–D12; Spearman test, *p* < 0.05 in all cases). Similarly, in the 5 s group, positive correlations were observed on D1, D4, D8, D10, D11, and D12. For the 10 s group, D1 and D2 were the only significant variables (Figure 3A and Supplementary Table 3). For the frequencies of the FHE and HE peaks, the 2 s group exhibited positive correlations across all 12 days (Spearman test, *p* < 0.05 in all cases), except for D3 (Figure 3B and Supplementary Table 4). Significant positive correlations were also found in the 5 s group except for D1, D8, and D9 on all days. In the 10 s group, the significant results were observed only in the early training period of D3 and D5, with a significant negative correlation on D1.

The correlation results indicate that the FHE and HE peaks were similar in groups with short and shorter CS durations (2 and 5 s) but diverged in the group with the longer CS duration (10 s). In particular, the two peaks nearly overlapped in the 2 s group.

### Group difference in FHE and HE peaks

In the results of one-way ANOVAs with the group factor (10, 5, and 2 s) using the Kruskal–Wallis H test, the FHE peaks exerted a significant group effect across the training days for both latency (in cases of D1, D2, D5, D7, D10, D11, and D12) and frequency (in cases of D2, D3, D4, D5, D7, D9, D10, and D12) (Figure 4A). *Post hoc* revealed significant differences mostly between the 10 and 2 s groups or between the 10 and 5 s groups for both latency and frequency, except for D2 in frequency (Dunn’s test, Bonferroni-corrected *p* < 0.05; Supplementary Tables 5, 6).

Meanwhile, one-way ANOVA results for the HE peaks revealed a pattern in which the significant group effect for latency on D5–D12 appeared to mirror the significance observed in frequency on D1–D4 (Figure 4B). *Post hoc* analysis for latency revealed significant differences between the 10 s group and other groups during training periods from D5 to D12 (Dunn’s test, Bonferroni-corrected *p* < 0.05; Supplementary Table 7). In the *post hoc* analysis for frequency, the group differences were significantly higher in the 2 s group than in other groups during the early training periods of D1 to D4 (Dunn’s test, Bonferroni-corrected *p* < 0.05; Supplementary Table 8).

Based on the results of the FHE and HE peaks, group differences in the FHE peaks were sporadic and irregular across the training days, whereas those in the HE peaks displayed a more consistent pattern. I concluded that the HE peak results reflected stimulus adaptation after D1 to D4, as differences in the HE peak latency between the longest and shortest CS groups persisted from D5 to D12. The results indicate that the HE peaks better explain the effect of CS duration than the FHE peaks.

### HE accuracy

**Figure 5:**
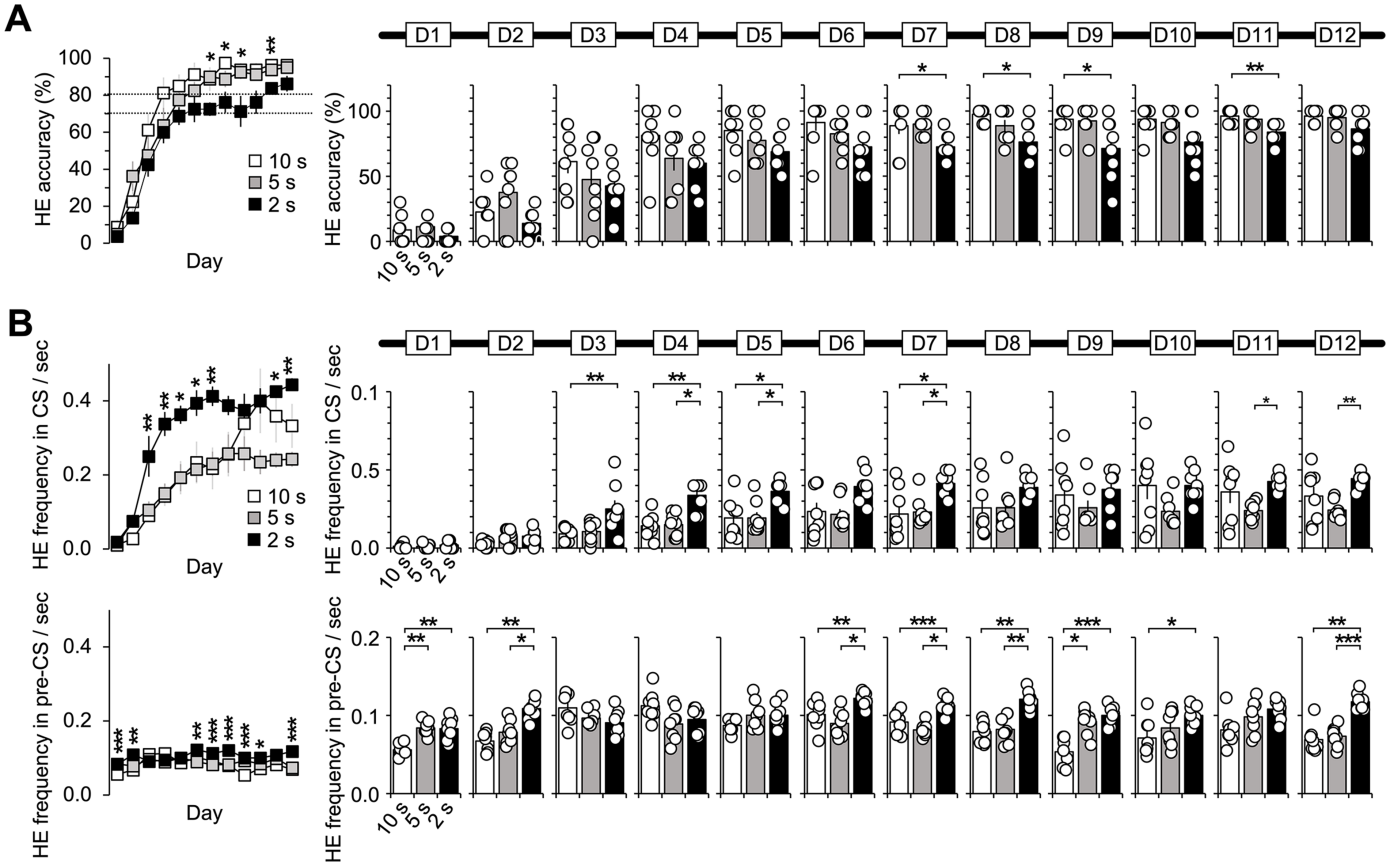
Basic properties of HE. **(A)** The one-way ANOVA results indicated that the group effect for HE accuracy was only present on D7, D8, D9, and D11 (Kruskal–Wallis H test, *p* < 0.05). However, all subjects eventually performed well on the tasks, with accuracy above the mean of 80%, regardless of the CS duration. **(B)** One-way ANOVA revealed a significant group effect for HE frequency during the CS period (D3–D5, D7, D11, and D12) (Kruskal–Wallis H test, *p* < 0.05). *Post hoc* analysis revealed that the 2 s group had a significantly higher frequency than the 5 and 10 s groups (Dunn’s test, *p* < 0.05, Bonferroni-corrected). During the pre-CS period, the group effect was significant on 8 days except for D3, D4, D5, and D11 (Kruskal–Wallis H test, *p* < 0.05). *Post hoc* analysis revealed that the 2 s group had higher frequencies than the 10 s group. D1 and D9 showed differences between the 10 and 5 s groups, while D2, D6, D7, D8, and D12 revealed differences between the 5 and 2 s groups (Dunn’s test, *p* < 0.05, Bonferroni-corrected). Supplementary Tables 9–11 also provide details of the statistical analyses. *, *p* < 0.05; **, *p* < 0.01; and ***, *p* < 0.001. Error bars denote 95% confidence intervals. HE, head entry; FHE, fastest HE; D, training day; CS, conditioned stimulus.

HE accuracy was determined based on whether the mice’s heads touched the food cup within the CS duration of 10, 5, and 2 s. The results of the one-way ANOVAs using the Kruskal–Wallis H test indicated significant group differences in HE accuracy on D7, D8, D9, and D11 (Figure 5A). *Post hoc* analyses revealed only differences between the 10 and 2 s groups (Dunn’s test, Bonferroni-corrected *p* < 0.05; Supplementary Table 9). However, no significant difference was discovered on the last day of D12. Overall, the subjects achieved high accuracy rates of more than 80%, regardless of the CS duration.

### HE frequency

HE frequency was calculated for both the CS and pre-CS periods, with the number of HEs normalized per 1 s for the three groups. Significant group differences were observed during the CS period in D3–D7, D11, and D12 (Figure 5B and Supplementary Table 10). Post hoc analysis revealed that the 2 s group exhibited significantly higher frequencies than the 5 and 10 s groups in D3, D4, D5, D7, D11, and D12 (Dunn’s test, Bonferroni-corrected *p* < 0.05). For the pre-CS period, one-way ANOVA results indicated significant group effects on 8 days except for D3, D4, D5, and D11 (Figure 5B and Supplementary Table 11). In *post hoc*, the 2 s group exhibited higher frequencies than the 10 and 5 s groups. The 10 and 2 s groups showed differences on the days except for D3, D4, D5, and D11. The 10 and 5 s groups showed differences on D1 and D9, and the 5 and 2 s groups differed on D2, D6, D7, D8, and D12 (Dunn’s test, Bonferroni-corrected *p* < 0.05). These findings show how CS duration affects the pre-CS resting state. Different CS durations most likely resulted in frequent or infrequent HEs from the subjects.

Additional analysis was conducted on baseline-corrected values of HE frequency in the CS and HE peak. The mean values of HE frequency and HE peak in the pre-CS period were used as the baselines, and each baseline effect was subtracted from HE frequency in the CS and HE peak. However, there was no prominent difference from the uncorrected results (Supplementary Figure 6 and Supplementary Tables 12–14). I concluded that the responses in the off-stimulus of pre-CS did not directly impact the HE peak and frequency in the on-stimulus of CS.

### Number of HE and correlation with accuracy

For each group, I counted the number of HEs in the CS period without normalizing per 1 s (as I did with HE frequency). As expected, subjects’ longer CS durations elicited a higher response rate (Supplementary Figure 7A). In the 10 s group, the HE occurrences increased to approximately three after D8. Contrarily, in the 2 s group, the HE occurrences remained below 1, whereas in the 5 s group, HE slightly surpassed 1. On D12, the mean and standard deviation values were 3.325 ± 1.676 in the 10 s group, 1.123 ± 0.285 in the 5 s group, and 0.888 ± 0.099 in the 2 s group. Significant group differences were observed between 10 and 2 s in D4 and D8–D12 (Dunn’s test, Bonferroni-corrected *p* < 0.05; Supplementary Table 15).

**Figure 6:**
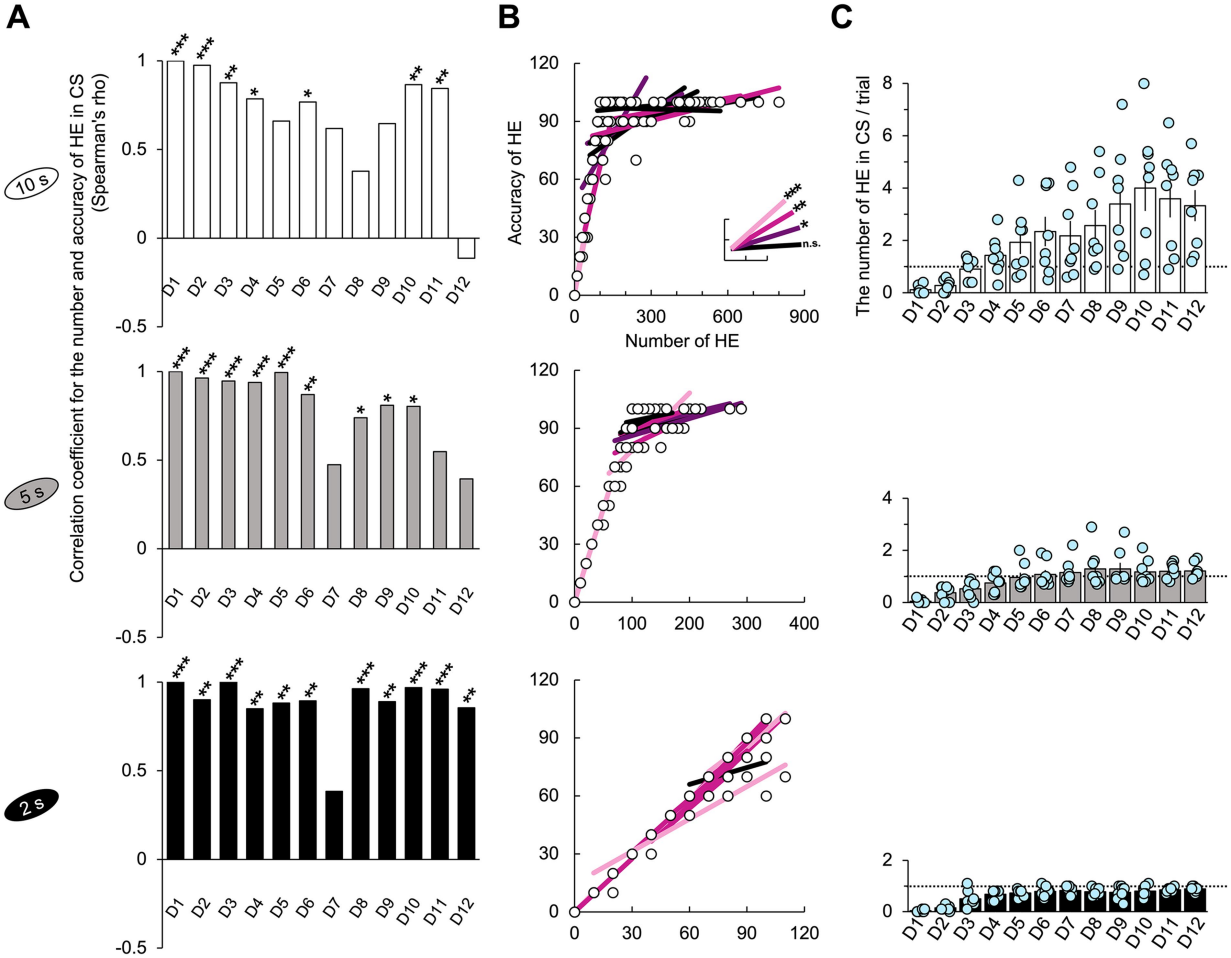
Number of HE and correlation with accuracy. **(A)** Significant positive correlations were observed between the number of HEs and accuracy across all groups (Spearman test, *p* < 0.05). For the 2 s group, these significances were consistent across all the training days except for D7. The nonparametric correlation was assessed using the Spearman’s test. **(B)** The graphs included the lines of best fit and data points corresponding to the correlation coefficients in Figure 6A and show the trend in the data for each day (Supplementary Figure 7B). The scale of the x-axis was determined by the maximum value observed across all the training days. In the case of the 2 s group, many data points overlap, resulting in a small representation of the 96 points collected from 8 subjects over 12 days. **(C)** The 10 s group exhibited the highest number of HEs. In the 2 s group, the HE occurrences were below 1, while in the 5 s group, HE slightly exceeded 1. The dotted line represents the number of HE that corresponds to a one-to-one matched relationship between the stimulus and response. Supplementary Tables 15, 16 also provide details of the statistical analyses. Significance levels are denoted as *, *p* < 0.05; **, *p* < 0.01; and ***, *p* < 0.001. Error bars represent 95% confidence intervals. Abbreviations used: HE for head entry, FHE for fastest head entry, D for training day, and CS for conditioned stimulus.

Next, I explored the relationship between the accuracy rate and the number of HEs per trial, which was normalized to a percentage value for an easier comparison with the accuracy rate. Each HE occurrence was scored as 10. The results were highly significant across all training days, except for D7 in the 2 s group (Spearman test, *p* < 0.05; Figure 6A and Supplementary Figure 7B). The 2 s group exhibited a linear trend in which the accuracy rate improved with increased HEs across all training days (Figure 6B and Supplementary Table 16), with the fewest HEs occurring less than once per trial (Figure 6C). However, the correlation results for the 5 and 10 s groups varied across training days and became less significant near the final day. The lack of significance in the correlation results for the 10 and 5 s groups could be attributed to the accuracy rate ceiling effect or an excess of HEs relative to the number of trials.

### Repetition of HE

In the 2 s group, the one-to-one matched relation may have occurred due to the mice’s restrained impulsivity for anticipated food reward, or it may simply reflect their natural tendency to momentarily pause after reaching the food cup. To explore these possibilities, I sorted the HEs for each trial chronologically and calculated the time intervals between them: from the CS onset to the first HE, from the first to the second HE, and between the subsequent HEs. I counted the number of mice involved in each HE order and the frequency with which responses occurred. If a mouse responded at least once in 10 trials, it was counted as one mouse in the order of that HE. For each order, the number of HEs was normalized to the percentile, with 80 responses to 80 trials equaling 100%. Because of incomplete successive observations of responses beyond the second HE for all subjects, statistical analysis could not be performed in this study.

**Figure 7:**
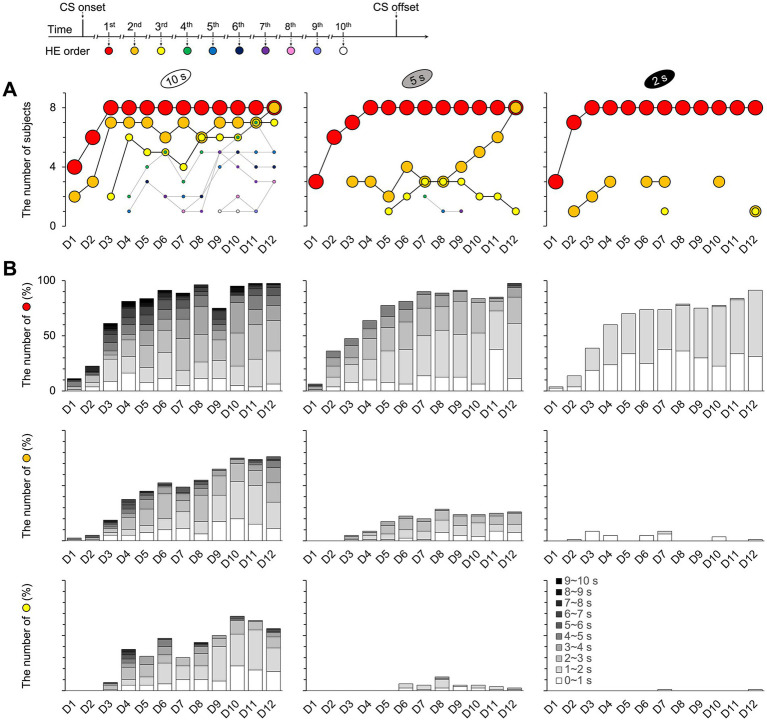
Number of repeating HEs and participating subjects per trial. The repetitions of HEs were observed up to 10 times across all trials and training days. **(A)** In the 10 s group, the responses were recorded until the tenth HE (Supplementary Figure 8B). Meanwhile, the maximum observed in the 5 s group was the seventh HE (Supplementary Figure 8B), while in the 2 s group, it was the third HE. The pattern of the first HE was consistent across all groups; however, differences were observed in the second and third HEs. Specifically, the second HE increased toward eight mice on D12 in both the 10 and 5 s groups but not for the 2 s group. The second HE of the 2 s group from D3 to D7, which had the same number of participating subjects as the 5 s group, was faster than the 5 s group, with intervals ranging from 0 to 1 s in **B**. However, it did not increase as the 5 s group toward D12. **(B)** The latencies between HEs varied with the CS durations. The interval latency was the longest in the 10 s group and the shortest in the 2 s group. See Supplementary Figure 8 for the details of the results from the fourth through the tenth HEs for the 10 and 5 s groups. HE, head entry; D, training day; CS, conditioned stimulus.

The maximum number of HE was 10 times across all trials and training days (Figure 7B and Supplementary Figure 8). In the 10 s group, one mouse responded 10 times on D9 and D10 (10th HE in Supplementary Figure 8B). The seventh HE in the 5 s group (7th HE in Supplementary Figure 8B) and the third HE in the 2 s group were the highest recorded (3rd HE in Figure 7B). The number of subjects who produced their first HE increased by D12 across all groups. In addition, up to eight mice of all subject numbers in both the 10 and 5 s groups produced the second HE by D12, indicating an association between the first and second HEs. However, this pattern did not appear in the 2 s group (Figure 7A). The second HE of the 2 s group from D3 to D7, which had a similar number of participating subjects to the 5 s group, was faster than that of the 5 s group, with intervals mostly ranging from 0 to 1 s. However, the number of subjects did not increase as they approached D12, unlike the 5 s group. Despite responding similarly during the 2 s CS period, the mice in this group exhibited fewer repeated HEs than the other groups.

These findings indicate that mice in the 2 s group stopped responding after the first HE during the CS period, waiting until the US was presented. Contrarily, the mice in the 10 and 5 s groups demonstrated more variable responses across training days, as evidenced by fluctuations in the correlation coefficients between the number of HEs and accuracy rate.

## Discussion

In the current data, it was difficult to determine which peak—FHE or HE—was closer to RT. A positive correlation between FHE and HE peaks resolved the dilemma of choosing between the two peaks. As the CS duration decreased, the FHE and HE peak latencies became more aligned, which resulted in fewer HE occurrences and increased monotony, similar to human responses (Kim et al., 2021; Kim et al., 2014). The basic properties of HE accuracy and frequency showed that the mice could reach and respond to the food cup despite varying CS durations. The HE accuracy scores exceeded 80% for all the 10-, 5-, and 2-s groups. The HE frequency in the short and shorter CS durations (5 and 2 s) was the same as in the long CS duration (10 s). The group differences in pre-CS did not affect the results in the CS period or HE peaks.

Timing is critical for investigating behavioral responses (Killeen and Fetterman, 1988). RT, also known as response time (Schouten and Bekker, 1967; Luce, 1986; Baayen and Milin, 2010; Welford et al., 1980), is an essential component in behavior analysis and serves as a marker reflecting each subject’s background (Welford, 1988), sensory and cognitive processes (Kim et al., 2014; Wendt et al., 2014; Krajbich et al., 2015; Iordanescu et al., 2010; Janata, 2004; Ahveninen et al., 2013; Rao et al., 2001; Sheth et al., 2012), and clinical perspective (Luck et al., 2006; Kaiser et al., 2008). RT is also associated with neural correlation across multiple brain regions (Binder et al., 2004; Lo and Wang, 2006; Wig et al., 2005). In addition to accuracy, the latency of RT accesses subjects’ task perception (Schouten and Bekker, 1967). In human studies (Luck et al., 2006; Binder et al., 2004), accuracy and RT are extracted and compared using the same data point. However, in animal studies, subjects may respond repeatedly, resulting in multiple RTs across multiple data points, forming a single accuracy rate. RT may be a more sensitive indicator than accuracy owing to their different mechanisms (Prinzmetal et al., 2005), which can greatly fluctuate depending on the researcher’s instructions. Subjects can be asked to slowly or quickly respond with little effect on accuracy scores. In this study, all three groups responded well to each stimulus (Figure 5A), but the FHE and HE peak latencies differed (Figure 4), indicating that different CS durations reflected the researchers’ instructions on response timing. Longer CS durations gave subjects more freedom in response timing, whereas shorter durations might have created a sense of pressure and unease in responding within a limited timeframe. Thus, the shorter CS duration of 2 s in this study represented good instruction for more sensitive RT, in which the FHE and HE peaks were nearly coincident in terms of latency and frequency, and accuracy rate and HE numbers were positively correlated over the training periods.

Temporal aspects of the experimental paradigm, including the interstimulus interval, intertrial interval, interval between CS and US, and CS duration, individually and collectively influence responses (Delamater and Holland, 2008; Thrailkill et al., 2020; Meltzer, 1986; Odling-Smee, 1975). This study focuses on the influence of CS duration on determining the CS–US interval in delay conditioning. In classical conditioning (Dickinson and Mackintosh, 1978; Millenson et al., 1977; Cooper, 1991), the time allotted for animal responses is dictated by the interval between the stimulus and the reward. Following the onset of the CS, the US, often food, directs the animals’ behaviors. Animals may delay their actions when faced with longer CS–US intervals. A short CS–US interval tends to elicit quicker responses, while a longer interval facilitates the thorough and secure acquisition of responses. More frequent responses might reflect behaviors associated with food addiction (Velazquez-Sanchez et al., 2014) and a profound understanding of the task (Celma-Miralles and Toro, 2020). Response latency and frequency are likely to show greater variance in distributions with longer CS durations, reflecting individual differences such as preferences, hesitation, and laziness, which diverge from typical human RT.

The FHE and HE peaks exhibited distinct patterns between the groups. The HE peak demonstrated the effect of different CS durations on latency and frequency across early to late training days, indicating a strong relationship with animals’ habituated behaviors for the stimulus (Groves and Thompson, 1970; de Boer et al., 1976; Thompson, 2009; Cevik, 2014; McSweeney et al., 1996). Contrarily, the FHE peak showed rarely significant group differences over several days. The frequencies serve as reliability indicators, reflecting the overlapping responses of individual subjects at specific time points (Harris and Carpenter, 2011; Austen and Sanderson, 2020; Jennings and Kirkpatrick, 2006). The nonsignificant group difference in the HE peak frequency after D5 (Figure 4B) corresponded to the saturation of the HE frequency (Figure 5B). The results of the HE peak indicate that all subjects in the three groups adapted to the CS duration and performed well within each CS period. However, the FHE peak results indicate that the response of the longer CS group was dispersed even within the subject’s data because of the low density of responses during the extended CS period compared to the shorter CS groups. The group differences in FHE and HE exhibited dissimilarity in latency and frequency. Based on the present findings, I proposed that the 2 s CS duration, which displays synchronized FHE and HE peaks in the current experimental paradigm, is the best CS duration for the attainment of unbiased results in mice behavior.

The 2 s group had the highest frequency during the pre-CS period, indicating that they checked the food cup more frequently during shorter CS periods. In addition, a linear relationship was observed between the HE frequency and CS duration during the pre-CS period. However, it is worth noting that behavior during the pre-CS period did not influence behavior during the on-stimulus period, as evidenced by the baseline-corrected results for the HE peak frequency and latency during CS. Despite having the highest number of HE occurrences during the CS period, the 10 s group had no significant differences in HE frequency compared to the 5 or 2 s groups. A linear relationship between CS duration and response (Holland, 1980; Thrailkill et al., 2020) was only observed between the 5 and the 2 s groups. Contrary to the linear trend in the pre-CS period, the non-linear trend in the CS period suggests that CS duration plays a different role during on- and off-stimulus conditions.

The HE frequency during the CS period in the 5 s group was significantly lower than that in the 2 s group. This indicates that the response pattern of the 5 and 2 s groups after the onset of the sound cue would most likely be similar. Interestingly, the latency and frequency of FHE and HE did not significantly differ between the 5 and 2 s groups, except for the HE peak frequency in D3 (Figure 4). The 5 and 2 s groups showed positive correlations between the FHE and HE peaks. However, the 2 s group, which exhibited a stronger correlation between the FHE and HE peaks, also showed a correlation between the number of HE and accuracy over the training days.

Meanwhile, the correlation results of the 5 s group indicated that the mice’s late-day behaviors were subtle and arbitrary, similar to those of the 10 s group. Furthermore, the 2 s group responded less than 1 per trial, whereas the 10 and 5 s groups responded with more than 1 HE. These findings suggest that the 5 and 2 s groups performed similarly; however, the mice in the 2 s group learned the one-to-one matched relationship between stimulus and response more effectively. Moreover, the results of the second HE indicated that the mice that produced the first HE repeated it at least once for any trial, but this was observed only in the 2 s group out of all the groups (Figure 7). These findings suggest that the mice in the 2 s group chose to stop responding during the remaining time until the US was presented after the first HE in the CS period. Contrarily, the 5 s group exhibited a steep linear increase in the number of subjects involved in the second HE, beginning with D8 and eventually reaching all subjects with D12, as did the 10 s group. The synchrony between the FHE and HE peaks, as well as between the number of HEs and accuracy in the 2 s group, was caused by the absence of the second HE. In the current data, the prediction for the US encompassed not only enhancement of response (Coddington and Dudman, 2018; Eshel et al., 2016; Anderson et al., 2020) but also both inhibition and promotion of response based on CS duration. I propose that the second HE, which is influenced by CS duration, can be interpreted as an indicator of impulsivity toward the US in the data.

The mice’s RT was comparable to that of humans based on the relationship between the FHE and HE peaks. In the paradigm, the shorter 2 s CS yielded a one-to-one matched relationship with less than 0.5 Hz response frequencies. However, if the data involves licking behavior with higher frequency responses in a head-fixed model, the relationship may change despite the presence of synchronization between the FHE and HE peaks. The one-to-one matched response would be tailored to the present paradigm. Rather than trace conditioning related to the memory-based process (Balsam, 1984), delay conditioning was applied in this study to elicit a response based on the stimulus duration without an interval of silence. These criteria should be confirmed in diverse paradigms, including studies using female mice and various species. In the results for the repetition of HE, the statistical analysis was not applied because the number of repeated HEs varied across subjects and orders. The analytical method must be quantified to establish the second HE as an indicator. In the current paradigm, the HE behavior serves not only as an operant response but also as a count recorded while the animal consumes the condensed milk. This results in the compounding of response time and reward collection latency into a single measure. This issue warrants further investigation in future studies.

**Figure 8:**
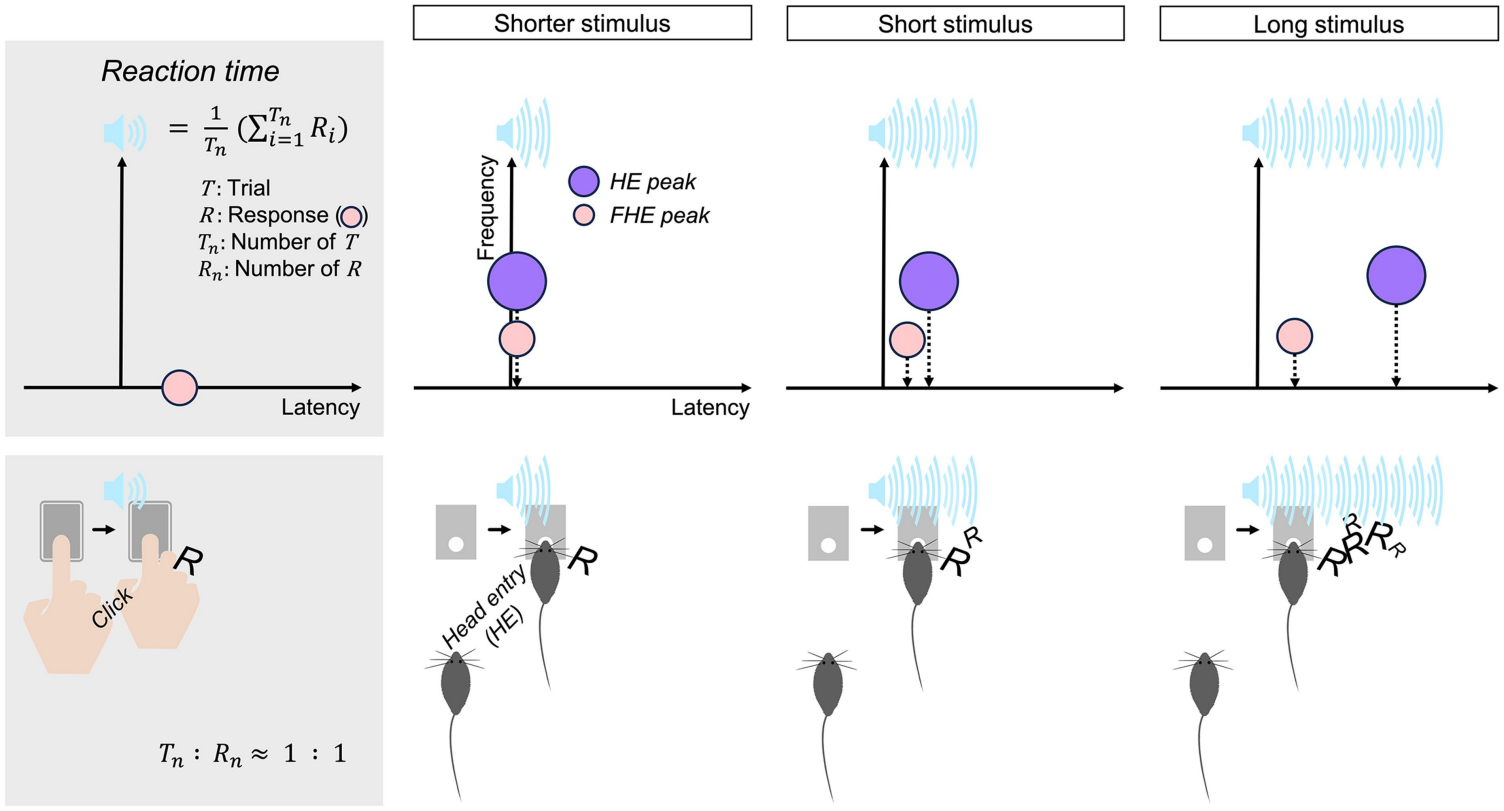
Reaction time model for mice. The harmonization of the HE and FHE peaks evoked by the shorter stimulus is associated with the humanlike response of mice. The number of responses to the shorter stimulus per trial is approximately 1, whereas the responses to the short and long stimuli are over 1, unlike humans. As for the number of responses, the computation of humanlike averaged RTs can be adapted only for the shorter stimulus. HE, head entry; FHE, fastest HE.

Nevertheless, the current results highlight that simply adjusting the CS duration can reduce repeated responses and encourage prompt responses similar to those observed in humans. The findings indicated how the variety of personalities among animals can be managed in terms of CS duration. Setting a time limit for responses can promote genuine participation without hesitation, thereby improving the validity and reliability of the behavioral data. Finally, I propose a novel model with quantifiable criteria for the evaluation of the stimulus duration in RT-related experimental paradigms regardless of apparatus and specific characteristics of the animals. This model is based on the harmony between FHE and HE peaks and the relationship between the number of HEs and accuracy (Figure 8). If animals are capable, the shorter CS durations, which can barely elicit one response per trial, may be preferable for eliciting humanlike responses with no repetition.

## Data Availability

The raw data supporting the conclusions of this article will be made available by the authors, without undue reservation.

## References

[ref1] Ahveninen J. Huang S. Nummenmaa A. Belliveau J. W. Hung A. Y. Jaaskelainen I. P.. (2013). Evidence for distinct human auditory cortex regions for sound location versus identity processing. Nat. Commun. 4:2585. doi: 10.1038/ncomms3585, PMID: 24121634 PMC3932554

[ref2] Altieri N. Townsend J. T. (2011). An assessment of behavioral dynamic information processing measures in audiovisual speech perception. Front. Psychol. 2:238. doi: 10.3389/fpsyg.2011.00238, PMID: 21980314 PMC3180170

[ref3] Anderson C. Von Keyserlingk M. Lidfors L. Weary D. (2020). Anticipatory behaviour in animals: a critical review. Anim. Welf. 29, 231–238. doi: 10.7120/09627286.29.3.231

[ref4] Austen J. M. Sanderson D. J. (2020). Cue duration determines response rate but not rate of acquisition of Pavlovian conditioning in mice. Q. J. Exp. Psychol. 73, 2026–2035. doi: 10.1177/1747021820937696, PMID: 32662337 PMC7583441

[ref5] Baayen R. H. Milin P. (2010). Analyzing reaction times. Int. J. Psychol. Res. 3, 12–28. doi: 10.21500/20112084.807

[ref6] Bale M. R. Bitzidou M. Pitas A. Brebner L. S. Khazim L. Anagnou S. T.. (2017). Learning and recognition of tactile temporal sequences by mice and humans. eLife 6:6. doi: 10.7554/eLife.27333, PMID: 28812976 PMC5559268

[ref7] Balodis I. M. Kober H. Worhunsky P. D. Stevens M. C. Pearlson G. D. Potenza M. N. (2012). Diminished frontostriatal activity during processing of monetary rewards and losses in pathological gambling. Biol. Psychiatry 71, 749–757. doi: 10.1016/j.biopsych.2012.01.006, PMID: 22336565 PMC3460522

[ref8] Balsam P. (1984). Relative time in trace conditioning. Ann. N. Y. Acad. Sci. 423, 211–227. doi: 10.1111/j.1749-6632.1984.tb23432.x, PMID: 6588787

[ref9] Binder J. R. Liebenthal E. Possing E. T. Medler D. A. Ward B. D. (2004). Neural correlates of sensory and decision processes in auditory object identification. Nat. Neurosci. 7, 295–301. doi: 10.1038/nn1198, PMID: 14966525

[ref10] Brewer G. A. (2011). Analyzing response time distributions. Z. Psychol. 219, 117–124. doi: 10.1027/2151-2604/a000056

[ref11] Butz M. V. Hoffmann J. (2002). Anticipations control behavior: animal behavior in an anticipatory learning classifier system. Adapt. Behav. 10, 75–96. doi: 10.1007/3-540-48104-4_1

[ref12] Celma-Miralles A. Toro J. M. (2020). Non-human animals detect the rhythmic structure of a familiar tune. Psychon. Bull. Rev. 27, 694–699. doi: 10.3758/s13423-020-01739-2, PMID: 32409921

[ref13] Cevik M. O. (2014). Habituation, sensitization, and Pavlovian conditioning. Front. Integr. Neurosci. 8:13. doi: 10.3389/fnint.2014.00013, PMID: 24574983 PMC3920081

[ref14] Clark R. E. Squire L. R. (1998). Classical conditioning and brain systems: the role of awareness. Science 280, 77–81. doi: 10.1126/science.280.5360.77, PMID: 9525860

[ref15] Coddington L. T. Dudman J. T. (2018). The timing of action determines reward prediction signals in identified midbrain dopamine neurons. Nat. Neurosci. 21, 1563–1573. doi: 10.1038/s41593-018-0245-7, PMID: 30323275 PMC6226028

[ref16] Cooper L. D. (1991). Temporal factors in classical-conditioning. Learn. Motiv. 22, 129–152. doi: 10.1016/0023-9690(91)90020-9

[ref17] de Boer E. Connor W. Davis H. Eggermont J. Galambos R. Geisler C.. (1976). Habituation and attention in the auditory system. Berlin Heidelberg: Springer, 343–389.

[ref18] Delamater A. R. Holland P. C. (2008). The influence of CS-US interval on several different indices of learning in appetitive conditioning. J. Exp. Psychol. Anim. Behav. Process. 34, 202–222. doi: 10.1037/0097-7403.34.2.202, PMID: 18426304 PMC2857343

[ref19] Dickinson A. Mackintosh N. J. (1978). Classical conditioning in animals. Annu. Rev. Psychol. 29, 587–612. doi: 10.1146/annurev.ps.29.020178.003103, PMID: 341791

[ref20] Eshel N. Roiser J. P. (2010). Reward and punishment processing in depression. Biol. Psychiatry 68, 118–124. doi: 10.1016/j.biopsych.2010.01.027, PMID: 20303067

[ref21] Eshel N. Tian J. Bukwich M. Uchida N. (2016). Dopamine neurons share common response function for reward prediction error. Nat. Neurosci. 19, 479–486. doi: 10.1038/nn.4239, PMID: 26854803 PMC4767554

[ref22] Esteves M. Moreira P. S. Sousa N. Leite-Almeida H. (2021). Assessing impulsivity in humans and rodents: taking the translational road. Front. Behav. Neurosci. 15:647922. doi: 10.3389/fnbeh.2021.647922, PMID: 34025369 PMC8134540

[ref23] Etzel B. C. Wright E. S. (1964). Effects of delayed reinforcement on response latency and acquisition learning under simultaneous and successive discrimination-learning in children. J. Exp. Child Psychol. 1, 281–293. doi: 10.1016/0022-0965(64)90043-8

[ref24] Grice G. R. Nullmeyer R. Spiker V. A. (1982). Human reaction-time - toward a general-theory. J. Exp. Psychol. Gen. 111, 135–153. doi: 10.1037/0096-3445.111.1.135

[ref25] Groves P. M. Thompson R. F. (1970). Habituation: a dual-process theory. Psychol. Rev. 77, 419–450. doi: 10.1037/h0029810, PMID: 4319167

[ref26] Harris J. A. Carpenter J. S. (2011). Response rate and reinforcement rate in Pavlovian conditioning. J. Exp. Psychol. Anim. Behav. Process. 37, 375–384. doi: 10.1037/a0024554, PMID: 21787098

[ref27] Holland P. C. (1980). CS-US interval as a determinant of the form of Pavlovian appetitive conditioned responses. J. Exp. Psychol. Anim. Behav. Process. 6, 155–174. doi: 10.1037/0097-7403.6.2.155, PMID: 7373230

[ref28] Iordanescu L. Grabowecky M. Franconeri S. Theeuwes J. Suzuki S. (2010). Characteristic sounds make you look at target objects more quickly. Atten. Percept. Psychophys. 72, 1736–1741. doi: 10.3758/APP.72.7.1736, PMID: 20952773 PMC3261720

[ref29] Janata P. (2004). When music tells a story. Nat. Neurosci. 7, 203–204. doi: 10.1038/nn0304-203, PMID: 14983178

[ref30] Jennings D. Kirkpatrick K. (2006). Interval duration effects on blocking in appetitive conditioning. Behav. Process. 71, 318–329. doi: 10.1016/j.beproc.2005.11.007, PMID: 16378697

[ref31] Johnson A. W. (2018). Examining the influence of CS duration and US density on cue-potentiated feeding through analyses of licking microstructure. Learn. Motiv. 61, 85–96. doi: 10.1016/j.lmot.2017.07.001, PMID: 30082927 PMC6075650

[ref32] Johnson S. A. Turner S. M. Santacroce L. A. Carty K. N. Shafiq L. Bizon J. L.. (2017). Rodent age-related impairments in discriminating perceptually similar objects parallel those observed in humans. Hippocampus 27, 759–776. doi: 10.1002/hipo.22729, PMID: 28342259 PMC5479708

[ref33] Kail R. (1991). Controlled and automatic processing during mental rotation. J. Exp. Child Psychol. 51, 337–347. doi: 10.1016/0022-0965(91)90081-3, PMID: 2072082

[ref34] Kaiser S. Roth A. Rentrop M. Friederich H. C. Bender S. Weisbrod M. (2008). Intra-individual reaction time variability in schizophrenia, depression and borderline personality disorder. Brain Cogn. 66, 73–82. doi: 10.1016/j.bandc.2007.05.007, PMID: 17604894

[ref35] Khamechian M. B. Kozyrev V. Treue S. Esghaei M. Daliri M. R. (2019). Routing information flow by separate neural synchrony frequencies allows for "functionally labeled lines" in higher primate cortex. Proc. Natl. Acad. Sci. USA 116, 12506–12515. doi: 10.1073/pnas.1819827116, PMID: 31147468 PMC6589668

[ref36] Killeen P. R. Fetterman J. G. (1988). A behavioral theory of timing. Psychol. Rev. 95, 274–295. doi: 10.1037/0033-295X.95.2.274, PMID: 3375401

[ref37] Kim C. H. Jin S. H. Kim J. S. Kim Y. Yi S. W. Chung C. K. (2021). Dissociation of connectivity for syntactic irregularity and perceptual ambiguity in musical chord stimuli. Front. Neurosci. 15:693629. doi: 10.3389/fnins.2021.693629, PMID: 34526877 PMC8435864

[ref38] Kim C. H. Lee S. Kim J. S. Seol J. Yi S. W. Chung C. K. (2014). Melody effects on ERANm elicited by harmonic irregularity in musical syntax. Brain Res. 1560, 36–45. doi: 10.1016/j.brainres.2014.02.045, PMID: 24607297

[ref39] Koelsch S. Schmidt B. H. Kansok J. (2002). Effects of musical expertise on the early right anterior negativity: an event-related brain potential study. Psychophysiology 39, 657–663. doi: 10.1111/1469-8986.3950657, PMID: 12236333

[ref40] Krajbich I. Bartling B. Hare T. Fehr E. (2015). Rethinking fast and slow based on a critique of reaction-time reverse inference. Nat. Commun. 6:7455. doi: 10.1038/ncomms8455, PMID: 26135809 PMC4500827

[ref41] Lo C. C. Wang X. J. (2006). Cortico-basal ganglia circuit mechanism for a decision threshold in reaction time tasks. Nat. Neurosci. 9, 956–963. doi: 10.1038/nn1722, PMID: 16767089

[ref42] Luce R. D. (1986). Response times: their role in inferring elementary mental organization. Oxford: Oxford University Press.

[ref43] Luck S. J. Fuller R. L. Braun E. L. Robinson B. Summerfelt A. Gold J. M. (2006). The speed of visual attention in schizophrenia: electrophysiological and behavioral evidence. Schizophr. Res. 85, 174–195. doi: 10.1016/j.schres.2006.03.040, PMID: 16713184

[ref44] Marco-Pallares J. Cucurell D. Cunillera T. García R. Andrés-Pueyo A. Münte T. F.. (2008). Human oscillatory activity associated to reward processing in a gambling task. Neuropsychologia 46, 241–248. doi: 10.1016/j.neuropsychologia.2007.07.016, PMID: 17804025

[ref45] McSweeney F. K. Hinson J. M. Cannon C. B. (1996). Sensitization-habituation may occur during operant conditioning. Psychol. Bull. 120, 256–271. doi: 10.1037/0033-2909.120.2.256

[ref46] McSweeney F. K. Murphy E. S. (2009). Sensitization and habituation regulate reinforcer effectiveness. Neurobiol. Learn. Mem. 92, 189–198. doi: 10.1016/j.nlm.2008.07.002, PMID: 18674628

[ref47] Mehraei G. Hickox A. E. Bharadwaj H. M. Goldberg H. Verhulst S. Liberman M. C.. (2016). Auditory brainstem response latency in noise as a marker of Cochlear Synaptopathy. J. Neurosci. 36, 3755–3764. doi: 10.1523/JNEUROSCI.4460-15.2016, PMID: 27030760 PMC4812134

[ref48] Meltzer D. (1986). Cs duration and reinforcement schedule effects on conditioned enhancement and positive conditioned suppression. Bull. Psychon. Soc. 24, 290–293. doi: 10.3758/BF03330144

[ref49] Meltzer D. (1988). Positive conditioned suppression after shifts in CS duration and US probability. Bull. Psychon. Soc. 26, 565–568. doi: 10.3758/BF03330123

[ref50] Meltzer D. Brahlek J. A. (1970). Conditioned suppression and conditioned enhancement with the same positive UCS: an effect of CS duration. J. Exp. Anal. Behav. 13, 67–73. doi: 10.1901/jeab.1970.13-67, PMID: 5415041 PMC1333658

[ref51] Millenson J. Kehoe E. J. Gormezano I. (1977). Classical conditioning of the rabbit's nictitating membrane response under fixed and mixed CS-US intervals. Learn. Motiv. 8, 351–366. doi: 10.1016/0023-9690(77)90057-1

[ref52] Novikov N. A. Nurislamova Y. M. Zhozhikashvili N. A. Kalenkovich E. E. Lapina A. A. Chernyshev B. V. (2017). Slow and fast responses: two mechanisms of trial outcome processing revealed by EEG oscillations. Front. Hum. Neurosci. 11:218. doi: 10.3389/fnhum.2017.00218, PMID: 28529478 PMC5418942

[ref53] Odling-Smee F. J. (1975). Background stimuli and the inter-stimulus interval during Pavlovian conditioning. Q. J. Exp. Psychol. 27, 387–392. doi: 10.1080/14640747508400498, PMID: 1196588

[ref54] Poulton E. C. (1950). Perceptual anticipation and reaction time. Q. J. Exp. Psychol. 2, 99–112. doi: 10.1080/17470215008416582

[ref55] Prinzmetal W. McCool C. Park S. (2005). Attention: reaction time and accuracy reveal different mechanisms. J. Exp. Psychol. Gen. 134, 73–92. doi: 10.1037/0096-3445.134.1.73, PMID: 15702964

[ref56] Rao S. M. Mayer A. R. Harrington D. L. (2001). The evolution of brain activation during temporal processing. Nat. Neurosci. 4, 317–323. doi: 10.1038/85191, PMID: 11224550

[ref57] Rosas J. M. Alonso G. (1996). Temporal discrimination and forgetting of CS duration in conditioned suppression. Learn. Motiv. 27, 43–57. doi: 10.1006/lmot.1996.0003

[ref58] Schouten J. F. Bekker J. A. (1967). Reaction time and accuracy. Acta Psychol. 27, 143–153. doi: 10.1016/0001-6918(67)90054-6, PMID: 6062205

[ref59] Schwarz-Stevens K. S. Cunningham C. L. (1993). Pavlovian conditioning of heart rate and body temperature with morphine: effects of CS duration. Behav. Neurosci. 107, 1039–1048. doi: 10.1037/0735-7044.107.6.1039, PMID: 8136057

[ref60] Sheth S. A. Mian M. K. Patel S. R. Asaad W. F. Williams Z. M. Dougherty D. D.. (2012). Human dorsal anterior cingulate cortex neurons mediate ongoing behavioural adaptation. Nature 488, 218–221. doi: 10.1038/nature11239, PMID: 22722841 PMC3416924

[ref61] Stefanics G. Hangya B. Hernádi I. Winkler I. Lakatos P. Ulbert I. (2010). Phase entrainment of Human Delta oscillations can mediate the effects of expectation on reaction speed. J. Neurosci. 30, 13578–13585. doi: 10.1523/JNEUROSCI.0703-10.2010, PMID: 20943899 PMC4427664

[ref62] Strickland J. A. Austen J. M. Sprengel R. Sanderson D. J. (2024). Knockout of NMDARs in CA1 and dentate gyrus fails to impair temporal control of conditioned behavior in mice. Hippocampus 34, 126–140. doi: 10.1002/hipo.23593, PMID: 38140716

[ref63] Thompson R. F. (2009). Habituation: a history. Neurobiol. Learn. Mem. 92, 127–134. doi: 10.1016/j.nlm.2008.07.011, PMID: 18703156 PMC2714193

[ref64] Thrailkill E. A. Todd T. P. Bouton M. E. (2020). Effects of conditioned stimulus (CS) duration, intertrial interval, and I/T ratio on appetitive Pavlovian conditioning. J. Exp. Psychol. Anim. Learn. Cogn. 46, 243–255. doi: 10.1037/xan0000241, PMID: 32175762 PMC7394717

[ref65] Tosun T. Gur E. Balci F. (2016). Mice plan decision strategies based on previously learned time intervals, locations, and probabilities. Proc. Natl. Acad. Sci. USA 113, 787–792. doi: 10.1073/pnas.1518316113, PMID: 26733674 PMC4725500

[ref66] Trott J. M. Hoffman A. N. Zhuravka I. Fanselow M. S. (2022). Conditional and unconditional components of aversively motivated freezing, flight and darting in mice. eLife 11:e75663. doi: 10.7554/eLife.75663, PMID: 35616523 PMC9173745

[ref67] Velazquez-Sanchez C. Ferragud A. Moore C. F. Everitt B. J. Sabino V. Cottone P. (2014). High trait impulsivity predicts food addiction-like behavior in the rat. Neuropsychopharmacology 39, 2463–2472. doi: 10.1038/npp.2014.98, PMID: 24776685 PMC4138758

[ref68] Welford A. T. (1988). Reaction time, speed of performance, and age. Ann. N. Y. Acad. Sci. 515, 1–17. doi: 10.1111/j.1749-6632.1988.tb32958.x, PMID: 3364878

[ref69] Welford W. Brebner J. M. Kirby N. (1980). Reaction times. Stanford, CA: Stanford University.

[ref70] Wendt D. Brand T. Kollmeier B. (2014). An eye-tracking paradigm for analyzing the processing time of sentences with different linguistic complexities. PLoS One 9:e100186. doi: 10.1371/journal.pone.0100186, PMID: 24950184 PMC4065036

[ref71] Wig G. S. Grafton S. T. Demos K. E. Kelley W. M. (2005). Reductions in neural activity underlie behavioral components of repetition priming. Nat. Neurosci. 8, 1228–1233. doi: 10.1038/nn1515, PMID: 16056222

