# Supplementary material for: Harmonization of the fastest and densest responses reflects humanlike reaction time in mice
Source: Front Neurosci. 2025 Jan 29;19:1501374. doi: 10.3389/fnins.2025.1501374 (PMC11813871; doi:10.3389/fnins.2025.1501374)
Supplement: Supplementary file 1 [file Data_Sheet_1.pdf]

Supplementary tables

Table number	Session	Group	Component	Mean	SEM	Statistical test	Z	P	*
Supplementary Table 1	D1	10 s	FHE	1.375	0.822	Wilcoxon signed-ranks test	-1.604	0.109	
			HE	2.875	1.217				
		5 s	FHE	4.000	1.225	Wilcoxon signed-ranks test	-1	0.317	
			HE	4.625	1.238				
		2 s	FHE	5.000	0.906	Wilcoxon signed-ranks test	-1	0.317	
			HE	3.750	0.773				
	D2	10 s	FHE	1.500	0.845	Wilcoxon signed-ranks test	0	1	
			HE	1.500	0.845				
		5 s	FHE	2.500	1.118	Wilcoxon signed-ranks test	0	1	
			HE	2.750	0.881				
		2 s	FHE	4.125	0.515	Wilcoxon signed-ranks test	-1	0.317	
			HE	3.500	0.567				
	D3	10 s	FHE	1.250	0.590	Wilcoxon signed-ranks test	-1.342	0.18	
			HE	2.375	0.706				
		5 s	FHE	3.375	1.401	Wilcoxon signed-ranks test	-1.342	0.18	
			HE	1.875	1.076				
		2 s	FHE	1.375	0.375	Wilcoxon signed-ranks test	0	1	
			HE	1.375	0.263				
	D4	10 s	FHE	1.125	0.515	Wilcoxon signed-ranks test	-1.476	0.14	
			HE	2.750	0.773				
		5 s	FHE	1.250	0.366	Wilcoxon signed-ranks test	0	1	
			HE	1.250	0.366				
		2 s	FHE	1.000	0.189	Wilcoxon signed-ranks test	0	1	
			HE	1.000	0.189				
	D5	10 s	FHE	1.750	0.250	Wilcoxon signed-ranks test	-2.207	0.027	*
			HE	4.875	0.990				
		5 s	FHE	1.500	0.420	Wilcoxon signed-ranks test	-1	0.317	
			HE	2.130	0.550				
		2 s	FHE	0.500	0.189	Wilcoxon signed-ranks test	0	1	
			HE	0.500	0.189				
	D6	10 s	FHE	1.500	0.327	Wilcoxon signed-ranks test	-1.342	0.18	
			HE	2.750	0.701				
		5 s	FHE	1.500	0.189	Wilcoxon signed-ranks test	-1.342	0.18	
			HE	2.125	0.441				
		2 s	FHE	0.875	0.227	Wilcoxon signed-ranks test	-1	0.317	
			HE	1.000	0.189				
	D7	10 s	FHE	1.750	0.164	Wilcoxon signed-ranks test	-2.06	0.039	*
			HE	3.500	0.627				
		5 s	FHE	0.875	0.227	Wilcoxon signed-ranks test	-1	0.317	
			HE	1.625	0.653				
		2 s	FHE	0.250	0.164	Wilcoxon signed-ranks test	-1.414	0.157	
			HE	0.500	0.189				
	D8	10 s	FHE	1.750	0.366	Wilcoxon signed-ranks test	-2.041	0.041	*
			HE	4.625	1.085				
		5 s	FHE	1.000	0.189	Wilcoxon signed-ranks test	-1	0.317	
			HE	1.125	0.227				
		2 s	FHE	0.750	0.250	Wilcoxon signed-ranks test	-1	0.317	
			HE	0.625	0.183				
	D9	10 s	FHE	1.500	0.535	Wilcoxon signed-ranks test	-2.032	0.042	*
			HE	4.625	0.754				
		5 s	FHE	1.250	0.250	Wilcoxon signed-ranks test	-1	0.317	
			HE	1.625	0.263				
		2 s	FHE	0.750	0.250	Wilcoxon signed-ranks test	-1	0.317	
			HE	0.875	0.227				
	D10	10 s	FHE	2.250	0.250	Wilcoxon signed-ranks test	-1.841	0.066	
			HE	4.500	0.802				
		5 s	FHE	1.250	0.164	Wilcoxon signed-ranks test	0	1	
			HE	1.250	0.164				
		2 s	FHE	0.750	0.164	Wilcoxon signed-ranks test	0	1	
			HE	0.750	0.164				
	D11	10 s	FHE	2.000	0.463	Wilcoxon signed-ranks test	-2.023	0.043	*
			HE	3.875	0.743				
		5 s	FHE	1.375	0.263	Wilcoxon signed-ranks test	-1	0.317	
			HE	1.500	0.189				
		2 s	FHE	0.625	0.183	Wilcoxon signed-ranks test	0	1	
			HE	0.625	0.183				
	D12	10 s	FHE	1.625	0.263	Wilcoxon signed-ranks test	-1.841	0.066	
			HE	3.500	0.906				
		5 s	FHE	1.250	0.164	Wilcoxon signed-ranks test	0	1	
			HE	1.250	0.164				
		2 s	FHE	0.630	0.180	Wilcoxon signed-ranks test	0	1	
			HE	0.630	0.180				

*P < 0.05, **P < 0.01, and ***P < 0.001

Table number	Session	Group	Component	Mean	SEM	Statistical test	Z	P	*
Supplementary Table 2	D1	10 s	FHE	1.625	0.183	Wilcoxon signed-ranks test	-1.734	0.083	
			HE	0.750	0.313				
		5 s	FHE	1.500	0.267	Wilcoxon signed-ranks test	-1.069	0.285	
			HE	1.000	0.267				
		2 s	FHE	2.250	0.250	Wilcoxon signed-ranks test	0	1	
			HE	2.250	0.313				
	D2	10 s	FHE	1.375	0.263	Wilcoxon signed-ranks test	-0.577	0.564	
			HE	1.250	0.313				
		5 s	FHE	1.750	0.164	Wilcoxon signed-ranks test	-0.577	0.564	
			HE	1.875	0.350				
		2 s	FHE	3.000	0.300	Wilcoxon signed-ranks test	-1.633	0.102	
			HE	3.500	0.400				
	D3	10 s	FHE	2.250	0.250	Wilcoxon signed-ranks test	-1.414	0.157	
			HE	2.500	0.189				
		5 s	FHE	2.500	0.327	Wilcoxon signed-ranks test	-1	0.317	
			HE	2.375	0.375				
		2 s	FHE	3.875	0.441	Wilcoxon signed-ranks test	-2.07	0.038	*
			HE	5.250	0.840				
	D4	10 s	FHE	2.375	0.263	Wilcoxon signed-ranks test	-1.857	0.063	
			HE	3.125	0.398				
		5 s	FHE	3.375	0.420	Wilcoxon signed-ranks test	-1.342	0.18	
			HE	3.750	0.559				
		2 s	FHE	4.500	0.423	Wilcoxon signed-ranks test	-1.89	0.059	
			HE	5.125	0.398				
	D5	10 s	FHE	2.625	0.324	Wilcoxon signed-ranks test	-2.214	0.027	*
			HE	4.250	0.726				
		5 s	FHE	3.875	0.693	Wilcoxon signed-ranks test	-1.414	0.157	
			HE	4.125	0.693				
		2 s	FHE	4.500	0.267	Wilcoxon signed-ranks test	-1	0.317	
			HE	4.625	0.324				
	D6	10 s	FHE	3.375	0.532	Wilcoxon signed-ranks test	-1.841	0.066	
			HE	4.750	0.840				
		5 s	FHE	4.500	0.707	Wilcoxon signed-ranks test	-1.342	0.18	
			HE	5.125	0.666				
		2 s	FHE	5.250	0.366	Wilcoxon signed-ranks test	-1.414	0.157	
			HE	5.500	0.327				
	D7	10 s	FHE	3.125	0.295	Wilcoxon signed-ranks test	-2.023	0.043	*
			HE	5.250	0.773				
		5 s	FHE	4.875	0.398	Wilcoxon signed-ranks test	-1	0.317	
			HE	5.000	0.327				
		2 s	FHE	5.000	0.535	Wilcoxon signed-ranks test	-1.857	0.063	
			HE	5.750	0.559				
	D8	10 s	FHE	3.875	0.479	Wilcoxon signed-ranks test	-2.032	0.042	*
			HE	5.750	0.861				
		5 s	FHE	4.500	0.463	Wilcoxon signed-ranks test	-1.633	0.102	
			HE	5.500	0.707				
		2 s	FHE	5.250	0.648	Wilcoxon signed-ranks test	0	1	
			HE	5.250	0.648				
	D9	10 s	FHE	3.125	0.295	Wilcoxon signed-ranks test	-2.384	0.017	*
			HE	6.250	0.921				
		5 s	FHE	4.875	0.441	Wilcoxon signed-ranks test	-1.342	0.180	
			HE	5.500	0.567				
		2 s	FHE	5.875	0.766	Wilcoxon signed-ranks test	-1.414	0.157	
			HE	6.125	0.718				
	D10	10 s	FHE	4.375	0.263	Wilcoxon signed-ranks test	-2.207	0.027	*
			HE	8.250	1.176				
		5 s	FHE	5.875	0.350	Wilcoxon signed-ranks test	-1	0.317	
			HE	6.125	0.227				
		2 s	FHE	5.625	0.532	Wilcoxon signed-ranks test	-1.414	0.157	
			HE	5.875	0.515				
	D11	10 s	FHE	4.500	0.189	Wilcoxon signed-ranks test	-2.226	0.026	*
			HE	7.125	0.833				
		5 s	FHE	5.125	0.515	Wilcoxon signed-ranks test	-2	0.046	*
			HE	5.625	0.565				
		2 s	FHE	6.000	0.535	Wilcoxon signed-ranks test	0	1	
			HE	6.000	0.535				
	D12	10 s	FHE	4.125	0.350	Wilcoxon signed-ranks test	-2.207	0.027	*
			HE	6.375	0.653				
		5 s	FHE	5.750	0.491	Wilcoxon signed-ranks test	-1.89	0.059	
			HE	6.375	0.498				
		2 s	FHE	6.500	0.500	Wilcoxon signed-ranks test	-1	0.317	
			HE	6.750	0.453				

*P < 0.05, **P < 0.01, and ***P < 0.001

Table number	Session	Group	Component	Mean	Statistical test	Correlation coffeficient	P	*
Supplementary Table 3	D1	10 s	FHE	1.375	Spearman test	0.863	0.006	**
			HE	2.875				
		5 s	FHE	4.000	Spearman test	0.900	0.002	**
			HE	4.625				
		2 s	FHE	5.000	Spearman test	0.300	0.470	
			HE	3.750				
	D2	10 s	FHE	1.500	Spearman test	1	0	***
			HE	1.500				
		5 s	FHE	2.500	Spearman test	-0.410	0.313	
			HE	2.750				
		2 s	FHE	4.125	Spearman test	0.405	0.320	
			HE	3.500				
	D3	10 s	FHE	1.250	Spearman test	0.181	0.669	
			HE	2.375				
		5 s	FHE	3.375	Spearman test	0.433	0.283	
			HE	1.875				
		2 s	FHE	1.375	Spearman test	0.304	0.463	
			HE	1.375				
	D4	10 s	FHE	1.125	Spearman test	-0.158	0.709	
			HE	2.750				
		5 s	FHE	1.250	Spearman test	1	0	***
			HE	1.250				
		2 s	FHE	1.000	Spearman test	1	0	***
			HE	1.000				
	D5	10 s	FHE	1.750	Spearman test	-0.033	0.938	
			HE	4.875				
		5 s	FHE	1.500	Spearman test	0.233	0.579	
			HE	2.130				
		2 s	FHE	0.500	Spearman test	1	0	***
			HE	0.500				
	D6	10 s	FHE	1.500	Spearman test	-0.052	0.903	
			HE	2.750				
		5 s	FHE	1.500	Spearman test	0.520	0.187	
			HE	2.125				
		2 s	FHE	0.875	Spearman test	0.819	0.013	*
			HE	1.000				
	D7	10 s	FHE	1.750	Spearman test	0	1	
			HE	3.500				
		5 s	FHE	0.875	Spearman test	0.236	0.573	
			HE	1.625				
		2 s	FHE	0.250	Spearman test	0.577	0.134	
			HE	0.500				
	D8	10 s	FHE	1.750	Spearman test	0.635	0.090	
			HE	4.625				
		5 s	FHE	1.000	Spearman test	0.819	0.013	*
			HE	1.125				
		2 s	FHE	0.750	Spearman test	0.926	0.001	**
			HE	0.625				
	D9	10 s	FHE	1.500	Spearman test	-0.348	0.398	
			HE	4.625				
		5 s	FHE	1.250	Spearman test	0.200	0.635	
			HE	1.625				
		2 s	FHE	0.750	Spearman test	0.843	0.009	**
			HE	0.875				
	D10	10 s	FHE	2.250	Spearman test	-0.491	0.217	
			HE	4.500				
		5 s	FHE	1.250	Spearman test	1	0	***
			HE	1.250				
		2 s	FHE	0.750	Spearman test	1	0	***
			HE	0.750				
	D11	10 s	FHE	2.000	Spearman test	0.098	0.818	
			HE	3.875				
		5 s	FHE	1.375	Spearman test	0.956	0.0002	***
			HE	1.500				
		2 s	FHE	0.625	Spearman test	1	0	***
			HE	0.625				
	D12	10 s	FHE	1.625	Spearman test	0.462	0.249	
			HE	3.500				
		5 s	FHE	1.250	Spearman test	1	0	***
			HE	1.250				
		2 s	FHE	0.630	Spearman test	1	0	***
			HE	0.630				

*$P < 0.05$, **$P < 0.01$, and ***$P < 0.001$

Table number	Session	Group	Component	Mean	Statistical test	Correlation coffeficient	P	*
Supplementary Table 4	D1	10 s	FHE	1.625	Spearman test	-0.852	0.007	**
			HE	0.750				
		5 s	FHE	1.500	Spearman test	-0.445	0.269	
			HE	1.000				
		2 s	FHE	2.250	Spearman test	0.803	0.016	*
			HE	2.250				
	D2	10 s	FHE	1.375	Spearman test	0.662	0.074	
			HE	1.250				
		5 s	FHE	1.750	Spearman test	0.811	0.015	*
			HE	1.875				
		2 s	FHE	3.000	Spearman test	0.775	0.024	*
			HE	3.500				
	D3	10 s	FHE	2.250	Spearman test	0.777	0.023	*
			HE	2.500				
		5 s	FHE	2.500	Spearman test	0.961	0.0001	***
			HE	2.375				
		2 s	FHE	3.875	Spearman test	0.614	0.105	
			HE	5.250				
	D4	10 s	FHE	2.375	Spearman test	0.687	0.060	
			HE	3.125				
		5 s	FHE	3.375	Spearman test	0.846	0.008	**
			HE	3.750				
		2 s	FHE	4.500	Spearman test	0.785	0.021	*
			HE	5.125				
	D5	10 s	FHE	2.625	Spearman test	0.864	0.006	**
			HE	4.250				
		5 s	FHE	3.875	Spearman test	0.963	0.0001	***
			HE	4.125				
		2 s	FHE	4.500	Spearman test	0.929	0.0008	***
			HE	4.625				
	D6	10 s	FHE	3.375	Spearman test	0.584	0.129	
			HE	4.750				
		5 s	FHE	4.500	Spearman test	0.723	0.043	*
			HE	5.125				
		2 s	FHE	5.250	Spearman test	0.883	0.004	**
			HE	5.500				
	D7	10 s	FHE	3.125	Spearman test	-0.032	0.940	
			HE	5.250				
		5 s	FHE	4.875	Spearman test	0.981	0.00002	***
			HE	5.000				
		2 s	FHE	5.000	Spearman test	0.916	0.001	**
			HE	5.750				
	D8	10 s	FHE	3.875	Spearman test	0.415	0.306	
			HE	5.750				
		5 s	FHE	4.500	Spearman test	0.417	0.304	
			HE	5.500				
		2 s	FHE	5.250	Spearman test	1	0	***
			HE	5.250				
	D9	10 s	FHE	3.125	Spearman test	0.051	0.905	
			HE	6.250				
		5 s	FHE	4.875	Spearman test	0.522	0.185	
			HE	5.500				
		2 s	FHE	5.875	Spearman test	0.975	0.00004	***
			HE	6.125				
	D10	10 s	FHE	4.375	Spearman test	-0.223	0.595	
			HE	8.250				
		5 s	FHE	5.875	Spearman test	0.841	0.009	**
			HE	6.125				
		2 s	FHE	5.625	Spearman test	0.975	0	***
			HE	5.875				
	D11	10 s	FHE	4.500	Spearman test	0.444	0.270	
			HE	7.125				
		5 s	FHE	5.125	Spearman test	0.950	0.0003	***
			HE	5.625				
		2 s	FHE	6.000	Spearman test	1	0	***
			HE	6.000				
	D12	10 s	FHE	4.125	Spearman test	0.119	0.780	
			HE	6.375				
		5 s	FHE	5.750	Spearman test	0.909	0.002	**
			HE	6.375				
		2 s	FHE	6.500	Spearman test	0.753	0.031	*
			HE	6.750				

*$P < 0.05$, **$P < 0.01$, and ***$P < 0.001$

Table number	Session	Group	Mean	SEM	Statistical test	Test Statistic	df	P	*	post-hoc test	Post hoc pair	Test Statistic	Bonferroni corrected P	*
Supplementary Table 5	D1	10 s	1.38	0.82	Kruskal–Wallis H test	6.362	2	0.042	*	Dunn's Test	10 s vs. 5 s	-6.125	0.235	
		5 s	4.00	1.22							10 s vs. 2 s	-8.500	0.044	*
		2 s	5.00	0.91							5 s vs. 2 s	-2.375	1.000	
	D2	10 s	1.50	0.85	Kruskal–Wallis H test	8.119	2	0.017	*	Dunn's Test	10 s vs. 5 s	-2.875	1.000	
		5 s	2.50	1.12							10 s vs. 2 s	-9.688	0.017	*
		2 s	4.13	0.52							5 s vs. 2 s	-6.813	0.153	
	D3	10 s	1.25	0.59	Kruskal–Wallis H test	1.485	2	0.476		Dunn's Test	10 s vs. 5 s			
		5 s	3.38	1.40							10 s vs. 2 s			
		2 s	1.38	0.38							5 s vs. 2 s			
	D4	10 s	1.13	0.52	Kruskal–Wallis H test	0.362	2	0.835		Dunn's Test	10 s vs. 5 s			
		5 s	1.25	0.37							10 s vs. 2 s			
		2 s	1.00	0.19							5 s vs. 2 s			
	D5	10 s	1.75	0.25	Kruskal–Wallis H test	0.834583901	2	0.011	*	Dunn's Test	10 s vs. 5 s	6.750	0.126	
		5 s	1.50	0.42							10 s vs. 2 s	9.750	0.010	*
		2 s	0.50	0.19							5 s vs. 2 s	3.000	1.000	
	D6	10 s	1.50	0.33	Kruskal–Wallis H test	3.78	2	0.151		Dunn's Test	10 s vs. 5 s			
		5 s	1.50	0.19							10 s vs. 2 s			
		2 s	0.88	0.23							5 s vs. 2 s			
	D7	10 s	1.75	0.16	Kruskal–Wallis H test	13.918	2	0.0009	***	Dunn's Test	10 s vs. 5 s	5.250	0.345	
		5 s	0.88	0.23							10 s vs. 2 s	12.375	0.0006	***
		2 s	0.25	0.16							5 s vs. 2 s	7.125	0.097	
	D8	10 s	1.75	0.37	Kruskal–Wallis H test	5.182	2	0.075		Dunn's Test	10 s vs. 5 s			
		5 s	1.00	0.19							10 s vs. 2 s			
		2 s	0.75	0.25							5 s vs. 2 s			
	D9	10 s	1.50	0.53	Kruskal–Wallis H test	1.735	2	0.420		Dunn's Test	10 s vs. 5 s			
		5 s	1.25	0.25							10 s vs. 2 s			
		2 s	0.75	0.25							5 s vs. 2 s			
	D10	10 s	2.25	0.25	Kruskal–Wallis H test	14.054	2	0.0009	***	Dunn's Test	10 s vs. 5 s	4.250	0.557	
		5 s	1.25	0.16							10 s vs. 2 s	11.875	0.0006	***
		2 s	0.75	0.16							5 s vs. 2 s	7.625	0.053	
	D11	10 s	2.00	0.46	Kruskal–Wallis H test	8.514	2	0.014	*	Dunn's Test	10 s vs. 5 s	6.625	0.131	
		5 s	1.38	0.26							10 s vs. 2 s	9.313	0.014	*
		2 s	0.63	0.18							5 s vs. 2 s	2.688	1.000	
	D12	10 s	1.63	0.26	Kruskal–Wallis H test	8.531	2	0.014	*	Dunn's Test	10 s vs. 5 s	5.875	0.163	
		5 s	1.25	0.16							10 s vs. 2 s	8.750	0.013	*
		2 s	0.63	0.18							5 s vs. 2 s	2.875	1.000	

*P < 0.05, **P < 0.01, and ***P < 0.001

Table number	Session	Group	Mean	SEM	Statistical test	Test Statistic	df	P	*	post-hoc test	Post hoc pair	Test Statistic	Bonferroni corrected P	*
Supplementary Table 6	D1	10 s	1.63	0.18	Kruskal–Wallis H test	4.902	2	0.086		Dunn's Test	10 s vs. 5 s			
		5 s	1.50	0.27							10 s vs. 2 s			
		2 s	2.25	0.25							5 s vs. 2 s			
	D2	10 s	1.38	0.26	Kruskal–Wallis H test	11.500	2	0.003	**	Dunn's Test	10 s vs. 5 s	-2.625	1.000	
		5 s	1.75	0.16							10 s vs. 2 s	-10.500	0.003	**
		2 s	3.00	0.33							5 s vs. 2 s	-7.875	0.044	*
	D3	10 s	2.25	0.25	Kruskal–Wallis H test	8.138	2	0.017	*	Dunn's Test	10 s vs. 5 s	-1.750	1.000	
		5 s	2.50	0.33							10 s vs. 2 s	-9.125	0.022	*
		2 s	3.88	0.44							5 s vs. 2 s	-7.375	0.090	
	D4	10 s	2.38	0.26	Kruskal–Wallis H test	10.054	2	0.007	**	Dunn's Test	10 s vs. 5 s	-5.375	0.339	
		5 s	3.38	0.42							10 s vs. 2 s	-10.750	0.005	**
		2 s	4.50	0.42							5 s vs. 2 s	-5.375	0.339	
	D5	10 s	2.63	0.32	Kruskal–Wallis H test	8.981	2	0.011	*	Dunn's Test	10 s vs. 5 s	-5.063	0.424	
		5 s	3.88	0.69							10 s vs. 2 s	-10.313	0.008	**
		2 s	4.50	0.27							5 s vs. 2 s	-5.250	0.381	
	D6	10 s	3.38	0.53	Kruskal–Wallis H test	5.013	2	0.082		Dunn's Test	10 s vs. 5 s			
		5 s	4.50	0.71							10 s vs. 2 s			
		2 s	5.25	0.37							5 s vs. 2 s			
	D7	10 s	3.13	0.30	Kruskal–Wallis H test	8.927	2	0.012	*	Dunn's Test	10 s vs. 5 s	-8.875	0.030	*
		5 s	4.88	0.40							10 s vs. 2 s	-8.938	0.028	*
		2 s	5.00	0.53							5 s vs. 2 s	-0.063	1.000	
	D8	10 s	3.88	0.48	Kruskal–Wallis H test	2.241	2	0.326		Dunn's Test	10 s vs. 5 s			
		5 s	4.50	0.46							10 s vs. 2 s			
		2 s	5.25	0.65							5 s vs. 2 s			
	D9	10 s	3.13	0.30	Kruskal–Wallis H test	10.405	2	0.006	**	Dunn's Test	10 s vs. 5 s	-8.250	0.053	
		5 s	4.88	0.44							10 s vs. 2 s	-10.688	0.006	**
		2 s	5.88	0.77							5 s vs. 2 s	-2.438	1.000	
	D10	10 s	4.38	0.26	Kruskal–Wallis H test	6.904	2	0.032	*	Dunn's Test	10 s vs. 5 s	-7.188	0.112	
		5 s	5.88	0.35							10 s vs. 2 s	-8.375	0.046	*
		2 s	5.63	0.53							5 s vs. 2 s	1.188	1.000	
	D11	10 s	4.50	0.19	Kruskal–Wallis H test	4.017	2	0.134		Dunn's Test	10 s vs. 5 s			
		5 s	5.13	0.52							10 s vs. 2 s			
		2 s	6.00	0.53							5 s vs. 2 s			
	D12	10 s	4.13	0.35	Kruskal–Wallis H test	9.962	2	0.007	**	Dunn's Test	10 s vs. 5 s	-7.625	0.082	
		5 s	5.75	0.49							10 s vs. 2 s	-10.563	0.007	**
		2 s	6.50	0.50							5 s vs. 2 s	-2.938	1.000	

*$P < 0.05$, **$P < 0.01$, and ***$P < 0.001$

Table number	Session	Group	Mean	SEM	Statistical test	Test Statistic	df	P	*	post-hoc test	Post hoc pair	Test Statistic	Bonferroni corrected P	*
Supplementary Table 7	D1	10 s	2.88	1.22	Kruskal–Wallis H test	1.549	2	0.461		Dunn's Test	10 s vs. 5 s			
		5 s	4.63	1.24							10 s vs. 2 s			
		2 s	3.75	0.77							5 s vs. 2 s			
	D2	10 s	1.50	0.85	Kruskal–Wallis H test	5.122	2	0.077		Dunn's Test	10 s vs. 5 s			
		5 s	2.75	0.88							10 s vs. 2 s			
		2 s	3.50	0.57							5 s vs. 2 s			
	D3	10 s	2.38	0.71	Kruskal–Wallis H test	1.468	2	0.480		Dunn's Test	10 s vs. 5 s			
		5 s	1.88	1.08							10 s vs. 2 s			
		2 s	1.38	0.26							5 s vs. 2 s			
	D4	10 s	2.75	0.77	Kruskal–Wallis H test	4.849	2	0.089		Dunn's Test	10 s vs. 5 s			
		5 s	1.25	0.37							10 s vs. 2 s			
		2 s	1.00	0.19							5 s vs. 2 s			
	D5	10 s	4.88	0.99	Kruskal–Wallis H test	15.765	2	0.0004	***	Dunn's Test	10 s vs. 5 s	7.313	0.102	
		5 s	2.13	0.55							10 s vs. 2 s	13.688	0.0002	***
		2 s	0.50	0.19							5 s vs. 2 s	6.375	0.194	
	D6	10 s	2.75	0.70	Kruskal–Wallis H test	7.498	2	0.024	*	Dunn's Test	10 s vs. 5 s	6.750	0.125	
		5 s	2.13	0.44							10 s vs. 2 s	8.625	0.028	*
		2 s	1.00	0.19							5 s vs. 2 s	1.875	1.000	
	D7	10 s	3.50	0.63	Kruskal–Wallis H test	14.557	2	0.0007	***	Dunn's Test	10 s vs. 5 s	5.063	0.416	
		5 s	1.63	0.65							10 s vs. 2 s	12.938	0.0005	***
		2 s	0.50	0.19							5 s vs. 2 s	7.875	0.064	
	D8	10 s	4.63	1.08	Kruskal–Wallis H test	12.069	2	0.002	**	Dunn's Test	10 s vs. 5 s	3.750	0.766	
		5 s	1.13	0.23							10 s vs. 2 s	11.250	0.002	**
		2 s	0.63	0.18							5 s vs. 2 s	7.500	0.069	
	D9	10 s	4.63	0.75	Kruskal–Wallis H test	16.145	2	0.0003	***	Dunn's Test	10 s vs. 5 s	4.500	0.565	
		5 s	1.63	0.26							10 s vs. 2 s	13.500	0.0002	***
		2 s	0.88	0.23							5 s vs. 2 s	9.000	0.026	*
	D10	10 s	4.50	0.80	Kruskal–Wallis H test	18.289	2	0.0001	***	Dunn's Test	10 s vs. 5 s	3.625	0.816	
		5 s	1.25	0.16							10 s vs. 2 s	13.625	0.0001	***
		2 s	0.75	0.16							5 s vs. 2 s	10.000	0.007	**
	D11	10 s	3.88	0.74	Kruskal–Wallis H test	16.653	2	0.0002	***	Dunn's Test	10 s vs. 5 s	6.500	0.162	
		5 s	1.50	0.19							10 s vs. 2 s	13.750	0.0001	***
		2 s	0.63	0.18							5 s vs. 2 s	7.250	0.095	
	D12	10 s	3.50	0.91	Kruskal–Wallis H test	12.427	2	0.002	**	Dunn's Test	10 s vs. 5 s	5.125	0.338	
		5 s	1.25	0.16							10 s vs. 2 s	11.375	0.0013	**
		2 s	0.63	0.18							5 s vs. 2 s	6.250	0.159	

*$P < 0.05$, **$P < 0.01$, and ***$P < 0.001$

Table number	*Session*	*Group*	*Mean*	*SEM*	*Statistical test*	*Test Statistic*	*df*	*P*	*	*post-hoc test*	*Post hoc pair*	*Test Statistic*	*Bonferroni corrected P*	*
Supplementary Table 8	***D1***	***10 s***	0.75	0.31	Kruskal–Wallis H test	8.808	2	0.012	*	Dunn's Test	***10 s vs. 5 s***	-1.750	1.000	
		5 s	1.00	0.27							***10 s vs. 2 s***	-9.500	0.016	*
		2 s	2.25	0.31							***5 s vs. 2 s***	-7.750	0.069	
	D2	***10 s***	1.25	0.31	Kruskal–Wallis H test	11.410	2	0.003	**	Dunn's Test	***10 s vs. 5 s***	-3.750	0.806	
		5 s	1.88	0.35							***10 s vs. 2 s***	-11.250	0.003	**
		2 s	3.50	0.42							***5 s vs. 2 s***	-7.500	0.081	
	D3	***10 s***	2.50	0.19	Kruskal–Wallis H test	10.724	2	0.005	**	Dunn's Test	***10 s vs. 5 s***	0.500	1.000	
		5 s	2.38	0.38							***10 s vs. 2 s***	-10.000	0.011	**
		2 s	5.25	0.84							***5 s vs. 2 s***	-9.500	0.017	**
	D4	***10 s***	3.13	0.40	Kruskal–Wallis H test	7.604	2	0.022	*	Dunn's Test	***10 s vs. 5 s***	-2.875	1.000	
		5 s	3.75	0.56							***10 s vs. 2 s***	-9.313	0.021	*
		2 s	5.13	0.40							***5 s vs. 2 s***	-6.438	0.188	
	D5	***10 s***	4.25	0.73	Kruskal–Wallis H test	1.044	2	0.593		Dunn's Test	***10 s vs. 5 s***			
		5 s	4.13	0.69							***10 s vs. 2 s***			
		2 s	4.63	0.32							***5 s vs. 2 s***			
	D6	***10 s***	4.75	0.84	Kruskal–Wallis H test	0.361	2	0.835		Dunn's Test	***10 s vs. 5 s***			
		5 s	5.13	0.67							***10 s vs. 2 s***			
		2 s	5.50	0.33							***5 s vs. 2 s***			
	D7	***10 s***	5.25	0.77	Kruskal–Wallis H test	0.835	2	0.542		Dunn's Test	***10 s vs. 5 s***			
		5 s	5.00	0.33							***10 s vs. 2 s***			
		2 s	5.75	0.56							***5 s vs. 2 s***			
	D8	***10 s***	5.75	0.86	Kruskal–Wallis H test	0.117	2	0.943		Dunn's Test	***10 s vs. 5 s***			
		5 s	5.50	0.71							***10 s vs. 2 s***			
		2 s	5.25	0.65							***5 s vs. 2 s***			
	D9	***10 s***	6.25	0.92	Kruskal–Wallis H test	0.282	2	0.869		Dunn's Test	***10 s vs. 5 s***			
		5 s	5.50	0.57							***10 s vs. 2 s***			
		2 s	6.13	0.72							***5 s vs. 2 s***			
	D10	***10 s***	8.25	1.18	Kruskal–Wallis H test	2.834	2	0.242		Dunn's Test	***10 s vs. 5 s***			
		5 s	6.13	0.23							***10 s vs. 2 s***			
		2 s	5.88	0.52							***5 s vs. 2 s***			
	D11	***10 s***	7.13	0.83	Kruskal–Wallis H test	1.871	2	0.392		Dunn's Test	***10 s vs. 5 s***			
		5 s	5.63	0.56							***10 s vs. 2 s***			
		2 s	6.00	0.53							***5 s vs. 2 s***			
	D12	***10 s***	6.38	0.65	Kruskal–Wallis H test	0.284	2	0.868		Dunn's Test	***10 s vs. 5 s***			
		5 s	6.38	0.50							***10 s vs. 2 s***			
		2 s	6.75	0.45							***5 s vs. 2 s***			

**P < 0.05, **P < 0.01, and ***P < 0.001*

Table number	Session	Group	Mean	SEM	Statistical test	Test Statistic	df	P	*	post-hoc test	Post hoc pair	Test Statistic	Bonferroni corrected P	*
Supplementary Table 9	D1	10 s	8.75	3.98	Kruskal–Wallis H test	1.989	2	0.370		Dunn's Test	10 s vs. 5 s			
		5 s	5.00	2.67							10 s vs. 2 s			
		2 s	3.75	1.83							5 s vs. 2 s			
	D2	10 s	22.50	5.90	Kruskal–Wallis H test	5.304	2	0.071		Dunn's Test	10 s vs. 5 s			
		5 s	36.25	8.65							10 s vs. 2 s			
		2 s	13.75	3.24							5 s vs. 2 s			
	D3	10 s	61.25	8.95	Kruskal–Wallis H test	2.004	2	0.367		Dunn's Test	10 s vs. 5 s			
		5 s	47.50	10.48							10 s vs. 2 s			
		2 s	42.50	6.48							5 s vs. 2 s			
	D4	10 s	81.25	8.33	Kruskal–Wallis H test	5.055	2	0.080		Dunn's Test	10 s vs. 5 s			
		5 s	63.75	9.44							10 s vs. 2 s			
		2 s	60.00	5.98							5 s vs. 2 s			
	D5	10 s	85.00	6.27	Kruskal–Wallis H test	3.715	2	0.156		Dunn's Test	10 s vs. 5 s			
		5 s	77.50	6.20							10 s vs. 2 s			
		2 s	68.75	4.79							5 s vs. 2 s			
	D6	10 s	91.25	6.39	Kruskal–Wallis H test	4.84	2	0.089		Dunn's Test	10 s vs. 5 s			
		5 s	82.50	4.53							10 s vs. 2 s			
		2 s	72.50	7.01							5 s vs. 2 s			
	D7	10 s	88.75	6.39	Kruskal–Wallis H test	7.509	2	0.023	*	Dunn's Test	10 s vs. 5 s	-0.6875	1.000	
		5 s	90.00	2.67							10 s vs. 2 s	-8.5	0.041	*
		2 s	72.50	3.66							5 s vs. 2 s	-7.8125	0.070	
	D8	10 s	97.50	1.64	Kruskal–Wallis H test	8.333	2	0.016	*	Dunn's Test	10 s vs. 5 s	-4.25	0.610	
		5 s	88.75	4.41							10 s vs. 2 s	-9.625	0.012	*
		2 s	76.25	5.65							5 s vs. 2 s	-5.375	0.323	
	D9	10 s	93.75	3.75	Kruskal–Wallis H test	6.918	2	0.031	*	Dunn's Test	10 s vs. 5 s	-1.0625	1.000	
		5 s	92.50	3.66							10 s vs. 2 s	-8.125	0.047	*
		2 s	71.25	8.33							5 s vs. 2 s	-7.0625	0.106	
	D10	10 s	93.75	3.75	Kruskal–Wallis H test	5.379	2	0.068		Dunn's Test	10 s vs. 5 s			
		5 s	91.25	2.95							10 s vs. 2 s			
		2 s	76.25	6.25							5 s vs. 2 s			
	D11	10 s	96.25	1.83	Kruskal–Wallis H test	9.692	2	0.008	**	Dunn's Test	10 s vs. 5 s	-2.0625	1.000	
		5 s	93.75	2.63							10 s vs. 2 s	-9.75	0.009	**
		2 s	83.75	2.63							5 s vs. 2 s	-7.6875	0.060	
	D12	10 s	96.25	1.83	Kruskal–Wallis H test	4.336	2	0.114		Dunn's Test	10 s vs. 5 s			
		5 s	95.00	2.67							10 s vs. 2 s			
		2 s	86.25	4.20							5 s vs. 2 s			

*P < 0.05, **P < 0.01, and ***P < 0.001

Table number	Session	Group	Mean	SEM	Statistical test	Test Statistic	df	P	*	post-hoc test	Post hoc pair	Test Statistic	Bonferroni corrected P	*
Supplementary Table 10	***D1***	***10 s***	0.011	0.005	Kruskal–Wallis H test	0.286	2	0.867		Dunn's Test	***10 s vs. 5 s***			
		5 s	0.010	0.005							***10 s vs. 2 s***			
		2 s	0.019	0.009							***5 s vs. 2 s***			
	D2	***10 s***	0.028	0.008	Kruskal–Wallis H test	5.546	2	0.062		Dunn's Test	***10 s vs. 5 s***			
		5 s	0.075	0.018							***10 s vs. 2 s***			
		2 s	0.075	0.016							***5 s vs. 2 s***			
	D3	***10 s***	0.090	0.015	Kruskal–Wallis H test	9.899	2	0.007	**	Dunn's Test	***10 s vs. 5 s***	-2.375	1.000	
		5 s	0.105	0.023							***10 s vs. 2 s***	-10.563	0.008	**
		2 s	0.250	0.055							***5 s vs. 2 s***	-8.188	0.060	
	D4	***10 s***	0.143	0.026	Kruskal–Wallis H test	12.39	2	0.002	**	Dunn's Test	***10 s vs. 5 s***	-0.875	1.000	
		5 s	0.150	0.027							***10 s vs. 2 s***	-11.125	0.005	**
		2 s	0.338	0.032							***5 s vs. 2 s***	-10.250	0.011	*
	D5	***10 s***	0.193	0.045	Kruskal–Wallis H test	9.224	2	0.010	*	Dunn's Test	***10 s vs. 5 s***	-1.000	1.000	
		5 s	0.193	0.036							***10 s vs. 2 s***	-9.688	0.017	*
		2 s	0.363	0.025							***5 s vs. 2 s***	-8.688	0.040	*
	D6	***10 s***	0.234	0.056	Kruskal–Wallis H test	6.199	2	0.045	*	Dunn's Test	***10 s vs. 5 s***	-1.500	1.000	
		5 s	0.215	0.035							***10 s vs. 2 s***	-6.750	0.168	
		2 s	0.394	0.035							***5 s vs. 2 s***	-8.250	0.058	
	D7	***10 s***	0.218	0.056	Kruskal–Wallis H test	9.386	2	0.009	**	Dunn's Test	***10 s vs. 5 s***	-1.438	1.000	
		5 s	0.230	0.033							***10 s vs. 2 s***	-10.000	0.014	*
		2 s	0.413	0.026							***5 s vs. 2 s***	-8.563	0.046	*
	D8	***10 s***	0.256	0.060	Kruskal–Wallis H test	4.883	2	0.087		Dunn's Test	***10 s vs. 5 s***			
		5 s	0.258	0.052							***10 s vs. 2 s***			
		2 s	0.388	0.026							***5 s vs. 2 s***			
	D9	***10 s***	0.339	0.076	Kruskal–Wallis H test	1.875	2	0.392		Dunn's Test	***10 s vs. 5 s***			
		5 s	0.258	0.047							***10 s vs. 2 s***			
		2 s	0.375	0.044							***5 s vs. 2 s***			
	D10	***10 s***	0.400	0.087	Kruskal–Wallis H test	5.554	2	0.062		Dunn's Test	***10 s vs. 5 s***			
		5 s	0.235	0.033							***10 s vs. 2 s***			
		2 s	0.400	0.034							***5 s vs. 2 s***			
	D11	***10 s***	0.359	0.071	Kruskal–Wallis H test	7.196	2	0.027	*	Dunn's Test	***10 s vs. 5 s***	-6.313	0.220	
		5 s	0.240	0.021							***10 s vs. 2 s***	-2.938	1.000	
		2 s	0.425	0.016							***5 s vs. 2 s***	-9.250	0.026	*
	D12	***10 s***	0.333	0.059	Kruskal–Wallis H test	9.378	2	0.009	**	Dunn's Test	***10 s vs. 5 s***	-4.000	0.761	
		5 s	0.243	0.020							***10 s vs. 2 s***	-6.625	0.176	
		2 s	0.444	0.018							***5 s vs. 2 s***	-10.625	0.007	**

**P < 0.05, **P < 0.01, and ***P < 0.001*

Table number	Session	Group	Mean	SEM	Statistical test	Test Statistic	df	P	*	post-hoc test	Post hoc pair	Test Statistic	Bonferroni corrected P	*
Supplementary Table 11	D1	10 s	0.056	0.003	Kruskal–Wallis H test	14.236	2	0.0008	***	Dunn's Test	10 s vs. 5 s	-11.438	0.004	**
		5 s	0.084	0.003							10 s vs. 2 s	-11.625	0.003	**
		2 s	0.083	0.005							5 s vs. 2 s	0.188	1.000	
	D2	10 s	0.067	0.004	Kruskal–Wallis H test	14.753	2	0.004	**	Dunn's Test	10 s vs. 5 s	-3.875	0.817	
		5 s	0.078	0.005							10 s vs. 2 s	-13.188	0.001	**
		2 s	0.109	0.005							5 s vs. 2 s	-9.313	0.025	*
	D3	10 s	0.110	0.007	Kruskal–Wallis H test	4.32	2	0.115		Dunn's Test	10 s vs. 5 s			
		5 s	0.097	0.003							10 s vs. 2 s			
		2 s	0.091	0.006							5 s vs. 2 s			
	D4	10 s	0.113	0.006	Kruskal–Wallis H test	4.789	2	0.091		Dunn's Test	10 s vs. 5 s			
		5 s	0.089	0.007							10 s vs. 2 s			
		2 s	0.095	0.006							5 s vs. 2 s			
	D5	10 s	0.087	0.003	Kruskal–Wallis H test	3.69	2	0.158		Dunn's Test	10 s vs. 5 s			
		5 s	0.100	0.006							10 s vs. 2 s			
		2 s	0.100	0.005							5 s vs. 2 s			
	D6	10 s	0.101	0.006	Kruskal–Wallis H test	11.806	2	0.003	**	Dunn's Test	10 s vs. 5 s	3.250	1.000	
		5 s	0.090	0.006							10 s vs. 2 s	-11.750	0.003	**
		2 s	0.122	0.003							5 s vs. 2 s	-8.500	0.048	*
	D7	10 s	0.092	0.005	Kruskal–Wallis H test	14.023	2	0.0009	***	Dunn's Test	10 s vs. 5 s	4.438	0.626	
		5 s	0.082	0.003							10 s vs. 2 s	-13.000	0.0007	***
		2 s	0.113	0.004							5 s vs. 2 s	-8.563	0.046	*
	D8	10 s	0.079	0.005	Kruskal–Wallis H test	14.954	2	0.0006	***	Dunn's Test	10 s vs. 5 s	-0.375	1.000	
		5 s	0.082	0.006							10 s vs. 2 s	-12.000	0.002	**
		2 s	0.121	0.004							5 s vs. 2 s	-11.625	0.003	**
	D9	10 s	0.053	0.006	Kruskal–Wallis H test	14.59	2	0.0007	***	Dunn's Test	10 s vs. 5 s	-10.063	0.013	*
		5 s	0.092	0.006							10 s vs. 2 s	-12.813	0.0009	***
		2 s	0.100	0.004							5 s vs. 2 s	-2.750	1.000	
	D10	10 s	0.071	0.008	Kruskal–Wallis H test	6.156	2	0.046	*	Dunn's Test	10 s vs. 5 s	-4.000	0.772	
		5 s	0.084	0.008							10 s vs. 2 s	-8.750	0.040	*
		2 s	0.100	0.004							5 s vs. 2 s	-4.750	0.536	
	D11	10 s	0.082	0.007	Kruskal–Wallis H test	5.959	2	0.051		Dunn's Test	10 s vs. 5 s			
		5 s	0.098	0.007							10 s vs. 2 s			
		2 s	0.108	0.004							5 s vs. 2 s			
	D12	10 s	0.069	0.006	Kruskal–Wallis H test	15.395	2	0.0005	***	Dunn's Test	10 s vs. 5 s	-2.375	1.000	
		5 s	0.073	0.005							10 s vs. 2 s	-13.000	0.0007	**
		2 s	0.118	0.004							5 s vs. 2 s	-10.625	0.008	***

*P < 0.05, **P < 0.01, and ***P < 0.001

Table number	Session	Group	Mean	SEM	Statistical test	Test Statistic	df	P	*	post-hoc test	Pair	Test Statistic	Bonferroni corrected P	*
Supplementary Table 12	D1	10 s	2.88	1.22	Kruskal–Wallis H test	1.549	2	0.461		Dunn's Test	10 s vs. 5 s			
		5 s	4.63	1.24							10 s vs. 2 s			
		2 s	3.75	0.77							5 s vs. 2 s			
	D2	10 s	1.50	0.85	Kruskal–Wallis H test	5.122	2	0.077		Dunn's Test	10 s vs. 5 s			
		5 s	2.75	0.88							10 s vs. 2 s			
		2 s	3.50	0.57							5 s vs. 2 s			
	D3	10 s	2.38	0.71	Kruskal–Wallis H test	1.468	2	0.480		Dunn's Test	10 s vs. 5 s			
		5 s	1.88	1.08							10 s vs. 2 s			
		2 s	1.38	0.26							5 s vs. 2 s			
	D4	10 s	2.75	0.77	Kruskal–Wallis H test	4.849	2	0.089		Dunn's Test	10 s vs. 5 s			
		5 s	1.25	0.37							10 s vs. 2 s			
		2 s	1.00	0.19							5 s vs. 2 s			
	D5	10 s	4.88	0.99	Kruskal–Wallis H test	15.765	2	0.0004	***	Dunn's Test	10 s vs. 5 s	-6.375	0.194	
		5 s	2.13	0.55							10 s vs. 2 s	-13.688	0.0002	***
		2 s	0.50	0.19							5 s vs. 2 s	-7.313	0.102	
	D6	10 s	2.75	0.70	Kruskal–Wallis H test	7.498	2	0.024	*	Dunn's Test	10 s vs. 5 s	-1.875	1.000	
		5 s	2.13	0.44							10 s vs. 2 s	-8.625	0.028	*
		2 s	1.00	0.19							5 s vs. 2 s	-6.750	0.125	
	D7	10 s	3.50	0.63	Kruskal–Wallis H test	14.557	2	0.0007	***	Dunn's Test	10 s vs. 5 s	-7.875	0.064	
		5 s	1.63	0.65							10 s vs. 2 s	-12.938	0.0005	***
		2 s	0.50	0.19							5 s vs. 2 s	-5.063	0.416	
	D8	10 s	4.63	1.08	Kruskal–Wallis H test	12.069	2	0.002	**	Dunn's Test	10 s vs. 5 s	-7.500	0.069	
		5 s	1.13	0.23							10 s vs. 2 s	-11.250	0.002	**
		2 s	0.63	0.18							5 s vs. 2 s	-3.750	0.766	
	D9	10 s	4.63	0.75	Kruskal–Wallis H test	16.145	2	0.0003	***	Dunn's Test	10 s vs. 5 s	-9.000	0.026	*
		5 s	1.63	0.26							10 s vs. 2 s	-13.500	0.0002	***
		2 s	0.88	0.23							5 s vs. 2 s	-4.500	0.565	
	D10	10 s	4.50	0.80	Kruskal–Wallis H test	18.289	2	0.0001	***	Dunn's Test	10 s vs. 5 s	-10.000	0.007	**
		5 s	1.25	0.16							10 s vs. 2 s	-13.625	0.0001	***
		2 s	0.75	0.16							5 s vs. 2 s	-3.625	0.816	
	D11	10 s	3.88	0.74	Kruskal–Wallis H test	16.653	2	0.0002	***	Dunn's Test	10 s vs. 5 s	-7.250	0.095	
		5 s	1.50	0.19							10 s vs. 2 s	-13.750	0.0001	***
		2 s	0.63	0.18							5 s vs. 2 s	-6.500	0.162	
	D12	10 s	3.50	0.91	Kruskal–Wallis H test	12.427	2	0.002	**	Dunn's Test	10 s vs. 5 s	-6.250	0.159	
		5 s	1.25	0.16							10 s vs. 2 s	-11.375	0.001	**
		2 s	0.63	0.18							5 s vs. 2 s	-5.125	0.338	

*P < 0.05, **P < 0.01, and ***P < 0.001

Table number	Session	Group	Mean	SEM	Statistical test	Test Statistic	df	P	*	post-hoc test	Pair	Test Statistic	Bonferroni corrected P	*
Supplementary Table 13	D1	10 s	0.48	0.29	Kruskal–Wallis H test	6.566	2	0.038	*	Dunn's Test	10 s vs. 5 s	-1.063	1.000	
		5 s	0.56	0.23							10 s vs. 2 s	-8.313	0.056	
		2 s	1.56	0.31							5 s vs. 2 s	-7.250	0.120	
	D2	10 s	0.79	0.27	Kruskal–Wallis H test	7.361	2	0.025	*	Dunn's Test	10 s vs. 5 s	-3.625	0.915	
		5 s	1.30	0.32							10 s vs. 2 s	-9.500	0.022	*
		2 s	2.58	0.44							5 s vs. 2 s	-5.875	0.289	
	D3	10 s	1.59	0.18	Kruskal–Wallis H test	11.37	2	0.003	**	Dunn's Test	10 s vs. 5 s	-0.375	1.000	
		5 s	1.57	0.36							10 s vs. 2 s	-10.500	0.009	**
		2 s	4.47	0.85							5 s vs. 2 s	-10.125	0.012	*
	D4	10 s	2.21	0.39	Kruskal–Wallis H test	8.994	2	0.011	*	Dunn's Test	10 s vs. 5 s	-3.625	0.915	
		5 s	2.96	0.50							10 s vs. 2 s	-10.438	0.009	**
		2 s	4.35	0.37							5 s vs. 2 s	-6.813	0.162	
	D5	10 s	3.49	0.71	Kruskal–Wallis H test	1.165	2	0.559		Dunn's Test	10 s vs. 5 s			
		5 s	3.28	0.66							10 s vs. 2 s			
		2 s	3.85	0.37							5 s vs. 2 s			
	D6	10 s	3.92	0.83	Kruskal–Wallis H test	0.635	2	0.728		Dunn's Test	10 s vs. 5 s			
		5 s	4.38	0.66							10 s vs. 2 s			
		2 s	4.66	0.34							5 s vs. 2 s			
	D7	10 s	4.50	0.73	Kruskal–Wallis H test	0.903	2	0.637		Dunn's Test	10 s vs. 5 s			
		5 s	4.31	0.30							10 s vs. 2 s			
		2 s	4.93	0.57							5 s vs. 2 s			
	D8	10 s	5.08	0.83	Kruskal–Wallis H test	0.466	2	0.792		Dunn's Test	10 s vs. 5 s			
		5 s	4.80	0.68							10 s vs. 2 s			
		2 s	4.34	0.66							5 s vs. 2 s			
	D9	10 s	5.77	0.90	Kruskal–Wallis H test	0.815	2	0.665		Dunn's Test	10 s vs. 5 s			
		5 s	4.74	0.53							10 s vs. 2 s			
		2 s	5.42	0.74							5 s vs. 2 s			
	D10	10 s	7.56	1.13	Kruskal–Wallis H test	3.518	2	0.172		Dunn's Test	10 s vs. 5 s			
		5 s	5.56	0.19							10 s vs. 2 s			
		2 s	5.16	0.54							5 s vs. 2 s			
	D11	10 s	6.33	0.83	Kruskal–Wallis H test	1.24	2	0.538		Dunn's Test	10 s vs. 5 s			
		5 s	4.97	0.61							10 s vs. 2 s			
		2 s	5.22	0.55							5 s vs. 2 s			
	D12	10 s	5.75	0.63	Kruskal–Wallis H test	0.035	2	0.983		Dunn's Test	10 s vs. 5 s			
		5 s	5.91	0.47							10 s vs. 2 s			
		2 s	5.87	0.47							5 s vs. 2 s			

*P < 0.05, **P < 0.01, and ***P < 0.001

Table number	Session	Group	Mean	SEM	Statistical test	Test Statistic	df	P	*	post-hoc test	Pair	Test Statistic	Bonferroni corrected P	*
Supplementary Table 14	D1	10 s	-0.04	0.01	Kruskal–Wallis H test	5.218	2	0.074		Dunn's Test	10 s vs. 5 s			
		5 s	-0.07	0.01							10 s vs. 2 s			
		2 s	-0.06	0.01							5 s vs. 2 s			
	D2	10 s	-0.04	0.01	Kruskal–Wallis H test	2.617	2	0.270		Dunn's Test	10 s vs. 5 s			
		5 s	0.00	0.02							10 s vs. 2 s			
		2 s	-0.03	0.02							5 s vs. 2 s			
	D3	10 s	-0.02	0.02	Kruskal–Wallis H test	10.151	2	0.006	**	Dunn's Test	10 s vs. 5 s	-2.563	1.000	
		5 s	0.01	0.02							10 s vs. 2 s	-10.750	0.007	**
		2 s	0.16	0.06							5 s vs. 2 s	-8.188	0.061	
	D4	10 s	0.03	0.02	Kruskal–Wallis H test	13.172	2	0.001	**	Dunn's Test	10 s vs. 5 s	-2.500	1.000	
		5 s	0.06	0.02							10 s vs. 2 s	-12.125	0.002	**
		2 s	0.24	0.03							5 s vs. 2 s	-9.625	0.019	*
	D5	10 s	0.11	0.05	Kruskal–Wallis H test	9.223	2	0.010	*	Dunn's Test	10 s vs. 5 s	-0.188	1.000	
		5 s	0.09	0.03							10 s vs. 2 s	-9.375	0.024	*
		2 s	0.26	0.03							5 s vs. 2 s	-9.188	0.028	*
	D6	10 s	0.13	0.06	Kruskal–Wallis H test	4.637	2	0.098		Dunn's Test	10 s vs. 5 s			
		5 s	0.13	0.03							10 s vs. 2 s			
		2 s	0.27	0.04							5 s vs. 2 s			
	D7	10 s	0.13	0.06	Kruskal–Wallis H test	7.949	2	0.019	*	Dunn's Test	10 s vs. 5 s	-1.438	1.000	
		5 s	0.15	0.03							10 s vs. 2 s	-9.250	0.026	*
		2 s	0.30	0.02							5 s vs. 2 s	-7.813	0.081	
	D8	10 s	0.18	0.06	Kruskal–Wallis H test	3.502	2	0.174		Dunn's Test	10 s vs. 5 s			
		5 s	0.18	0.05							10 s vs. 2 s			
		2 s	0.27	0.03							5 s vs. 2 s			
	D9	10 s	0.29	0.07	Kruskal–Wallis H test	2.641	2	0.267		Dunn's Test	10 s vs. 5 s			
		5 s	0.17	0.05							10 s vs. 2 s			
		2 s	0.28	0.05							5 s vs. 2 s			
	D10	10 s	0.33	0.08	Kruskal–Wallis H test	4.74	2	0.093		Dunn's Test	10 s vs. 5 s			
		5 s	0.15	0.03							10 s vs. 2 s			
		2 s	0.30	0.04							5 s vs. 2 s			
	D11	10 s	0.28	0.07	Kruskal–Wallis H test	7.408	2	0.025	*	Dunn's Test	10 s vs. 5 s	-6.875	0.154	
		5 s	0.14	0.02							10 s vs. 2 s	-2.375	1.000	
		2 s	0.32	0.02							5 s vs. 2 s	-9.250	0.026	*
	D12	10 s	0.26	0.06	Kruskal–Wallis H test	5.959	2	0.051		Dunn's Test	10 s vs. 5 s			
		5 s	0.17	0.02							10 s vs. 2 s			
		2 s	0.33	0.02							5 s vs. 2 s			

**P < 0.05, **P < 0.01, and ***P < 0.001*

Table number	Session	Group	Mean	SEM	Statistical test	Test Statistic	df	P	*	post-hoc test	Pair	Test Statistic	Bonferroni corrected P	*
Supplementary Table 15	***D1***	***10 s***	0.11	0.05	Kruskal–Wallis H test	0.84	2	0.657		Dunn's Test	***10 s vs. 5 s***			
		5 s	0.05	0.03							***10 s vs. 2 s***			
		2 s	0.04	0.02							***5 s vs. 2 s***			
	D2	***10 s***	0.28	0.08	Kruskal–Wallis H test	3.698	2	0.157		Dunn's Test	***10 s vs. 5 s***			
		5 s	0.38	0.09							***10 s vs. 2 s***			
		2 s	0.15	0.03							***5 s vs. 2 s***			
	D3	***10 s***	0.90	0.15	Kruskal–Wallis H test	4.436	2	0.109		Dunn's Test	***10 s vs. 5 s***			
		5 s	0.53	0.12							***10 s vs. 2 s***			
		2 s	0.50	0.11							***5 s vs. 2 s***			
	D4	***10 s***	1.43	0.26	Kruskal–Wallis H test	7.656	2	0.022	*	Dunn's Test	***10 s vs. 5 s***	-1.500	0.094	
		5 s	0.75	0.13							***10 s vs. 2 s***	-9.000	0.029	*
		2 s	0.68	0.06							***5 s vs. 2 s***	-7.500	1.000	
	D5	***10 s***	1.93	0.45	Kruskal–Wallis H test	5.844	2	0.054		Dunn's Test	***10 s vs. 5 s***			
		5 s	0.96	0.18							***10 s vs. 2 s***			
		2 s	0.73	0.05							***5 s vs. 2 s***			
	D6	***10 s***	2.34	0.56	Kruskal–Wallis H test	5.879	2	0.053		Dunn's Test	***10 s vs. 5 s***			
		5 s	1.08	0.17							***10 s vs. 2 s***			
		2 s	0.79	0.07							***5 s vs. 2 s***			
	D7	***10 s***	2.18	0.56	Kruskal–Wallis H test	5.594	2	0.061		Dunn's Test	***10 s vs. 5 s***			
		5 s	1.15	0.16							***10 s vs. 2 s***			
		2 s	0.83	0.05							***5 s vs. 2 s***			
	D8	***10 s***	2.56	0.60	Kruskal–Wallis H test	12.009	2	0.002	**	Dunn's Test	***10 s vs. 5 s***	-6.000	0.236	
		5 s	1.29	0.26							***10 s vs. 2 s***	-12.188	0.002	**
		2 s	0.78	0.05							***5 s vs. 2 s***	-6.188	0.264	
	D9	***10 s***	3.39	0.76	Kruskal–Wallis H test	12.431	2	0.002	**	Dunn's Test	***10 s vs. 5 s***	-6.063	0.221	
		5 s	1.29	0.23							***10 s vs. 2 s***	-12.313	0.001	**
		2 s	0.75	0.09							***5 s vs. 2 s***	-6.250	0.248	
	D10	***10 s***	4.00	0.87	Kruskal–Wallis H test	10.581	2	0.005	**	Dunn's Test	***10 s vs. 5 s***	-5.250	0.236	
		5 s	1.18	0.16							***10 s vs. 2 s***	-11.438	0.003	**
		2 s	0.80	0.07							***5 s vs. 2 s***	-6.188	0.408	
	D11	***10 s***	3.59	0.71	Kruskal–Wallis H test	13.851	2	0.00098	***	Dunn's Test	***10 s vs. 5 s***	-6.813	0.225	
		5 s	1.20	0.10							***10 s vs. 2 s***	-13.063	0.0006	***
		2 s	0.85	0.03							***5 s vs. 2 s***	-6.250	0.157	
	D12	***10 s***	3.33	0.59	Kruskal–Wallis H test	17.786	2	0.0001	***	Dunn's Test	***10 s vs. 5 s***	-7.313	0.098	
		5 s	1.21	0.10							***10 s vs. 2 s***	-14.813	0.0001	***
		2 s	0.89	0.04							***5 s vs. 2 s***	-7.500	0.112	

**P < 0.05, **P < 0.01, and ***P < 0.001*

Table number	Session	Group	Component	Mean	Statistical test	Correlation coffeficient	P	*
Supplementary Table 16	D1	10 s	Number of HE*	11.25	Spearman test	1	0	***
			HE accuracy	8.75				
		5 s	Number of HE*	5.00	Spearman test	1.000	0.0001	***
			HE accuracy	5.00				
		2 s	Number of HE*	3.75	Spearman test	1	0	***
			HE accuracy	3.75				
	D2	10 s	Number of HE*	27.50	Spearman test	0.975	0.00004	***
			HE accuracy	22.50				
		5 s	Number of HE*	37.50	Spearman test	0.963	0.0001	***
			HE accuracy	36.25				
		2 s	Number of HE*	15.00	Spearman test	0.900	0.002	**
			HE accuracy	13.75				
	D3	10 s	Number of HE*	90.00	Spearman test	0.877	0.004	**
			HE accuracy	61.25				
		5 s	Number of HE*	52.50	Spearman test	0.946	0.0004	***
			HE accuracy	47.50				
		2 s	Number of HE*	50.00	Spearman test	1	0	***
			HE accuracy	42.50				
	D4	10 s	Number of HE*	142.50	Spearman test	0.786	0.021	*
			HE accuracy	81.25				
		5 s	Number of HE*	75.00	Spearman test	0.938	0.0006	***
			HE accuracy	63.75				
		2 s	Number of HE*	67.50	Spearman test	0.850	0.007	**
			HE accuracy	60.00				
	D5	10 s	Number of HE*	192.50	Spearman test	0.661	0.074	
			HE accuracy	85.00				
		5 s	Number of HE*	96.25	Spearman test	0.994	0.000001	***
			HE accuracy	77.50				
		2 s	Number of HE*	72.50	Spearman test	0.882	0.004	**
			HE accuracy	68.75				
	D6	10 s	Number of HE*	233.75	Spearman test	0.768	0.026	*
			HE accuracy	91.25				
		5 s	Number of HE*	107.50	Spearman test	0.870	0.005	**
			HE accuracy	82.50				
		2 s	Number of HE*	78.75	Spearman test	0.894	0.003	**
			HE accuracy	72.50				
	D7	10 s	Number of HE*	217.50	Spearman test	0.619	0.102	
			HE accuracy	88.75				
		5 s	Number of HE*	115.00	Spearman test	0.474	0.235	
			HE accuracy	90.00				
		2 s	Number of HE*	82.50	Spearman test	0.384	0.348	
			HE accuracy	72.50				
	D8	10 s	Number of HE*	256.25	Spearman test	0.378	0.356	
			HE accuracy	97.50				
		5 s	Number of HE*	128.75	Spearman test	0.739	0.036	*
			HE accuracy	88.75				
		2 s	Number of HE*	77.50	Spearman test	0.963	0.0001	***
			HE accuracy	76.25				
	D9	10 s	Number of HE*	338.75	Spearman test	0.646	0.083	
			HE accuracy	93.75				
		5 s	Number of HE*	128.75	Spearman test	0.809	0.015	*
			HE accuracy	92.50				
		2 s	Number of HE*	75.00	Spearman test	0.890	0.003	**
			HE accuracy	71.25				
	D10	10 s	Number of HE*	400.00	Spearman test	0.866	0.005	**
			HE accuracy	93.75				
		5 s	Number of HE*	117.50	Spearman test	0.803	0.016	*
			HE accuracy	91.25				
		2 s	Number of HE*	80.00	Spearman test	0.969	0.00007	***
			HE accuracy	76.25				
	D11	10 s	Number of HE*	358.75	Spearman test	0.845	0.008	**
			HE accuracy	96.25				
		5 s	Number of HE*	120.00	Spearman test	0.548	0.160	
			HE accuracy	93.75				
		2 s	Number of HE*	85.00	Spearman test	0.960	0.00016	***
			HE accuracy	83.75				
	D12	10 s	Number of HE*	332.50	Spearman test	-0.113	0.789	
			HE accuracy	96.25				
		5 s	Number of HE*	121.25	Spearman test	0.394	0.334	
			HE accuracy	95.00				
		2 s	Number of HE*	88.75	Spearman test	0.855	0.007	**
			HE accuracy	86.25				

Number of HE = Number of HE * 100*

**P* < 0.05, ***P* < 0.01, and ****P* < 0.001
